# Exploring the Efficacy of Low-Temperature Plasmas on Oral Biofilms: A Scoping Review

**DOI:** 10.3390/medsci13020079

**Published:** 2025-06-18

**Authors:** Carson C. Davis, Fabrízio Dias Panariello, Beatriz Panariello

**Affiliations:** 1 School of Dental Medicine, Lake Erie College of Osteopathic Medicine, Bradenton, FL 34211, USA; 2 School of Medical Sciences, São Leopoldo Mandic, Campinas 13045-755, SP, Brazil

**Keywords:** low-temperature plasma, cold plasma, non-thermal plasma, oral biofilm, dental plaque, microbial biofilm, oral cavity, dentistry, oral health, oral tissues, plasma gases, biofilms, mouth

## Abstract

The rise of antibiotic resistance and the limitations of conventional therapies for managing biofilm-related oral infections highlight the urgent need for novel solutions, with low-temperature plasma (LTP) emerging as a promising alternative due to its potent antimicrobial effects, tissue-safety, and reduced risk of fostering resistance. This scoping review investigates the efficacy of LTP application for the management of oral biofilms associated with dental caries, peri-implantitis, endodontic infections, and oral candidiasis. This review was conducted in accordance with the PRISMA-ScR guidelines and registered with the Open Science Framework (OSF). Studies were identified through comprehensive searches of PubMed/MEDLINE, EBSCO (Medline Ultimate and e-journals), and Google Scholar, with no publication date restrictions, and were supplemented by manual reference screening. Eligible studies included original research, published in English, examining LTP’s effectiveness in oral biofilms. After systematically screening the literature, 51 studies were included in this scoping review, comprising mostly in vitro research, alongside ex vivo, in situ, and clinical studies. Data extraction revealed LTP’s broad-spectrum antimicrobial potential and promising clinical implications for dentistry. This review highlights key findings, identifies research gaps, and underscores the therapeutic potential of LTP in managing complex oral biofilm-related infections.

## 1. Introduction

Oral biofilms, complex microbial communities embedded in a protective extracellular matrix, are central to the pathogenesis of numerous dental conditions, including dental caries, peri-implantitis, endodontic infections, and oral candidiasis [1,2,3]. These biofilms, often comprising diverse bacterial and fungal species such as Streptococcus mutans, Porphyromonas gingivalis, Enterococcus faecalis, and Candida albicans, adhere to dental and mucosal surfaces and are notoriously resistant to conventional antimicrobial therapies [1,2,4,5,6,7].

The growing prevalence of antibiotic resistance, coupled with the limitations of traditional treatments, such as chlorhexidine for periodontal management, sodium hypochlorite for root canal infections, and nystatin or metronidazole for oral candidiasis, underscores the urgent need for innovative approaches to effectively manage biofilm-related oral infections [6,7]. Low-temperature plasma (LTP) has emerged as a promising technology for addressing these challenges, thanks to its strong antimicrobial properties, minimal thermal impact on biological tissues for most of the LTP systems, and lower likelihood of contributing to antimicrobial resistance [8,9].

LTP generates a complex mixture of reactive oxygen and nitrogen species (RONS), charged particles, and ultraviolet radiation, which collectively disrupt microbial cell membranes, DNA, RNA, proteins, and enzymes, induce oxidative stress, and impair biofilm structural integrity [8,9,10]. LTP can be generated through various electrical discharges, including dielectric barrier discharge (DBD), plasma jets, plasma needles, plasma pencils, glow discharge, and corona discharge systems [8,9,10,11,12,13]. Unlike thermal plasmas, LTP operates at near-ambient temperatures, making it suitable for applications in sensitive biological environments, such as the oral cavity, without causing thermal damage to tissues or dental materials [9,10,11,12,13]. Recent studies have demonstrated the efficacy of LTP in reducing the viability of pathogenic and opportunistic microorganisms in oral biofilms across various in vitro, in situ, ex vivo, and clinical models.

Despite the growing interest in LTP as a potential treatment for oral biofilm-related infections, there remains a lack of comprehensive synthesis of the available evidence on its efficacy. The variability in study designs, methodologies, and outcomes across different in vitro, ex vivo, in situ, and clinical studies creates a need to map and summarize the existing literature. A scoping review is particularly suitable for this purpose, as it will allow us to identify key themes, research gaps, and areas where further investigation is needed. By consolidating the available evidence, this review will provide a clearer understanding of LTP’s therapeutic potential for oral biofilms, inform future research, and guide clinical applications.

In this review, the term low-temperature plasma (LTP) refers to plasmas that operate at near-ambient temperatures and are therefore suitable for biological applications. This includes both non-thermal plasmas (NTPs), where energy is primarily carried by electrons, rather than by thermal motion, resulting in gas temperatures close to ambient, and cold atmospheric plasmas (CAPs), a subset of non-thermal plasmas that operate at atmospheric pressure without significant heating of the surrounding gas. While these terms are sometimes used interchangeably in the biomedical literature [9], we recognize the physical distinctions and have adopted a consistent terminology for clarity.

## 2. Materials and Methods

### 2.1. Study Design

This scoping review was conducted following the Preferred Reporting Items for Systematic Reviews and Meta-Analyses Extension for Scoping Reviews (PRISMA-ScR) guidelines. The review protocol was registered with the Open Science Framework (OSF) database (DOI 10.17605/OSF.IO/ZXY39) to ensure transparency and adherence to a standardized methodology.

### 2.2. Population, Intervention, Comparison, Outcome (PICO)

- Population: Individuals or samples with oral biofilms related to dental caries, peri-implantitis, endodontic infections, or oral candidiasis;
- Intervention: Low-temperature plasma;
- Comparison: No treatment, conventional antimicrobial treatments, or alternative disinfection/sterilization methods;
- Outcomes: Reduction or inhibition of oral biofilms, effectiveness of LTP, mechanisms of action, and therapeutic potential.

### 2.3. Eligibility Criteria

Inclusion criteria:
  - Original research articles investigating the use of LTP in relation to oral biofilms or within the field of dentistry;
  - Studies providing data on the effectiveness of LTP in biofilm treatment or the mechanisms of action;
  - Articles reporting outcomes related to biofilm inhibition or reduction, antimicrobial activity, or clinical relevance.
Exclusion criteria:
  - Review articles, commentary papers, posters, conference abstracts, or books;
  - Studies unrelated to oral biofilms, LTP, or dentistry in general;
  - Research not available in English.

### 2.4. Search Strategy

Electronic database searches were performed using PubMed/MEDLINE, EBSCO (Medline Ultimate, e-Journals), and Google Scholar. The search strategy incorporated a combination of MeSH terms (e.g., “Plasma Gases,” “Biofilms,” “Mouth”) and keywords (e.g., “cold plasma,” “non-thermal plasma,” “low-temperature plasma,” “oral biofilm,” “dental plaque,” “microbial biofilm,” “oral cavity,” “dentistry,” “oral health,” and “oral tissues”), along with Boolean operators (AND, OR) to refine the results. No restrictions were placed on the publication date. Only papers published in English were included in the search. In addition to database searches, a hand search of the reference lists of selected papers was conducted to identify additional relevant studies. The full search strategy is detailed in Table S1.

### 2.5. Screening and the Selection Process

All identified records were independently screened by two reviewers (B.P. and F.D.P.) based on predefined inclusion and exclusion criteria. Titles, abstracts, and full-text articles were assessed systematically. In cases of disagreement, a third independent reviewer (C.D.) mediated discussions to reach a consensus. The final selection of studies was documented.

### 2.6. Data Extraction and Charting

Data were systematically extracted from the included studies using a standardized charting form, capturing key information such as the type of study conducted (e.g., in vitro, in vivo, in situ, or clinical), a detailed description of the specific LTP technology applied (e.g., device type and gas composition), the microorganisms present within the biofilm (e.g., *P. gingivalis*, *S. mutans*, *C. albicans*), and the main findings of each study. These findings encompassed biofilm reduction, antimicrobial activity, and broader implications. Extracted data were synthesized to evaluate trends, identify evidence gaps, and summarize the relevance of LTP in managing oral biofilms. For the discussion, studies were categorized based on the type of biofilms they examined, including those related to cariogenic biofilms, peri-implantitis, endodontic infections, and candidal biofilms.

## 3. Results

**Figure 1:**
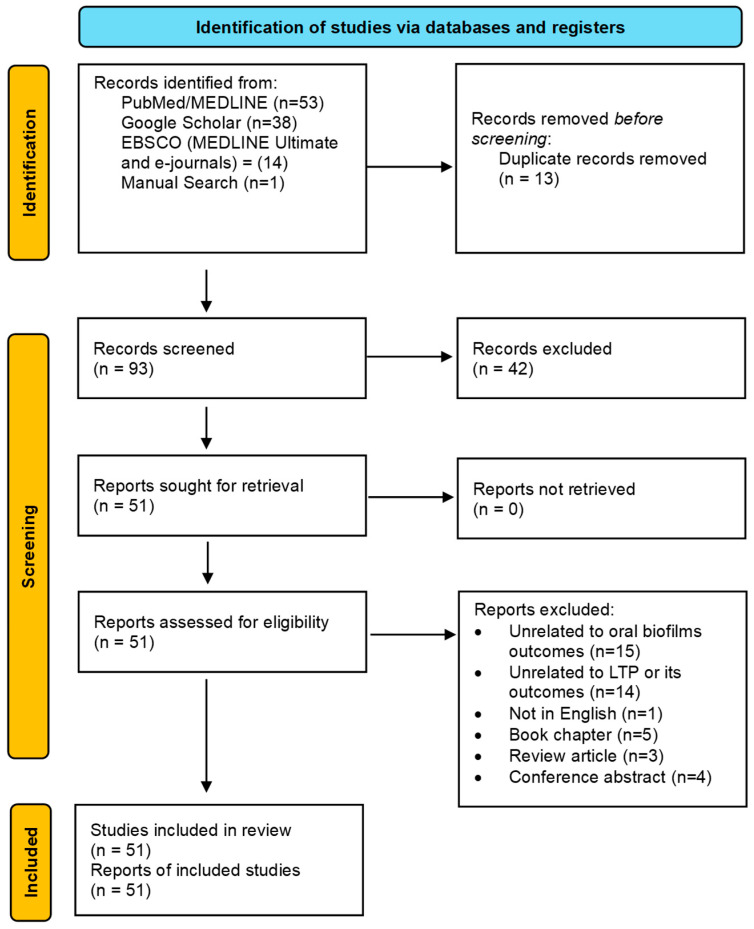
Flowchart of the studies screened, retrieved, and included in this scoping review.

A total of 51 studies were selected for this scoping review, as depicted in the PRISMA flowchart [14] (Figure 1). The publication years ranged from 2011 to 2025. A detailed summary of the selected studies, including study type, funding sources, biofilm characteristics, type of LTP device used, application details, key results, and conclusions, can be found in Table S2. Out of the 51 studies, the majority [11,12,13,15,16,17,18,19,20,21,22,23,24,25,26,27,28,29,30,31,32,33,34,35,36,37,38,39,40,41,42,43,44,45,46,47,48,49,50,51,52,53,54] were in vitro investigations; one study combined in vitro and ex vivo methodologies [55], two studies combined in vitro and in vivo approaches [56,57], one was ex vivo [58], two were clinical studies [59,60], and two were in situ studies [61,62]. The studies were grouped according to the type of biofilms they investigated: 15 studies focused on cariogenic biofilms [15,16,17,19,20,21,26,30,35,39,47,52,53,55,57], which included single-species, multispecies, or saliva-derived biofilms; and 21 studies explored biofilms associated with peri-implantitis [11,13,22,25,27,31,32,36,37,42,43,44,45,46,49,51,56,59,60,61,62], primarily formed by *P. gingivalis*, either alone or in combination with other species, or biofilms obtained from saliva or subgingival plaque samples from peri-implantitis patients. Nine studies concentrated on endodontic biofilms [18,23,24,28,33,40,41,50,58], predominantly formed by *E. faecalis*. Six studies focused on biofilms related to oral candidiasis, mainly formed by *C. albicans*, or antibiotic-resistant strains of bacteria [12,29,34,38,48,54]. A total of 42 studies were excluded from this review for the following reasons: they were unrelated to oral biofilm outcomes (n = 15), unrelated to LTP or its outcomes (n = 14), not published in English (n = 1), book chapters (n = 5), review articles (n = 3), or conference abstracts (n = 4). The excluded papers are provided in Table S3.

**Figure 2:**
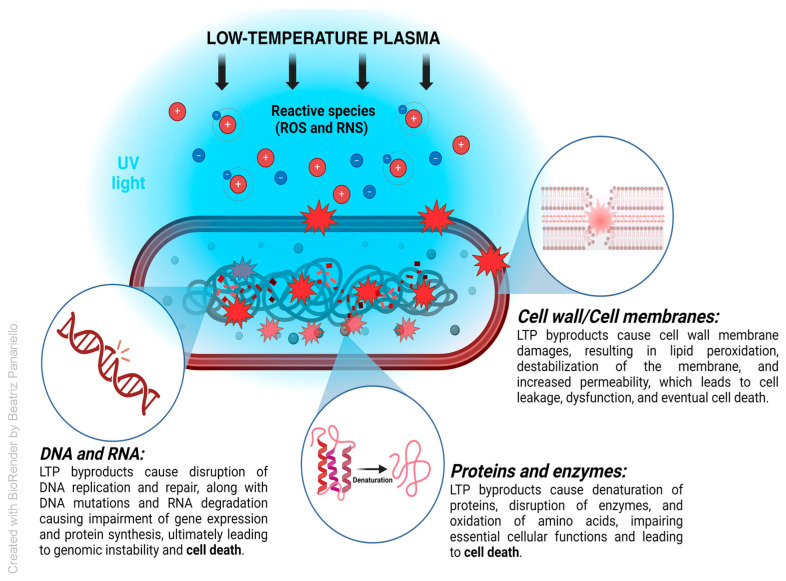
Schematic representation of the antimicrobial mechanisms of LTP on a bacterial cell, showing the generation of reactive oxygen species (ROS) and reactive nitrogen species (RNS), charged particles, and ultraviolet radiation, which collectively disrupt microbial membranes, damage nucleic acids and proteins, induce oxidative stress, and degrade biofilm structure.

Most studies utilized either dielectric barrier discharge devices or atmospheric pressure plasma jets, which differ in terms of energy delivery, exposure geometry, and reactive species generation. Helium and argon were the most commonly used carrier gases, often supplemented with oxygen. The plasma–biofilm interaction is further influenced by treatment time and distance from the target surface. These variables were reported in detail for each study in Table S2, and collectively, they demonstrate that LTP’s efficacy stems from a multifactorial mechanism shaped by both chemical and physical components tailored to the experimental setup. Figure 2 summarizes the mechanism of action of low-temperature plasmas.

## 4. Discussion

### 4.1. LTP on Cariogenic Biofilms

The antimicrobial efficacy of LTP treatments has been consistently demonstrated across various in vitro and ex vivo models of cariogenic biofilms, ranging from single-species *Streptococcus mutans* biofilms to complex multispecies communities that more closely mimic clinical conditions. Across the reviewed studies, consistent patterns of biofilm reduction, membrane damage, and enhanced antimicrobial effects—particularly when LTP is combined with conventional agents—highlight its potential as a target to cariogenic biofilms.

In mono-species models, LTP treatment resulted in rapid bacterial inactivation and significant reductions in viability. Yang et al. (2011) [17] achieved complete *S. mutans* elimination in under 15 s using a low-temperature argon plasma brush, with scanning electron microscopy (SEM) confirming extensive cell deformation and intracellular leakage. Blumhagen et al. (2014) [21] and Hong et al. (2019) [57] similarly observed substantial viability reductions, reporting decreases up to 2.5 log_10_ CFU following plasma treatment. Figueira et al. (2021) [35] documented a significant reduction in *S. mutans* biofilm viability, reinforcing LTP’s potent antimicrobial effects. Liu et al. (2017) [26] further support these findings, showing over 97% viability reduction in *S. mutans* biofilms following two minutes of plasma exposure. The role of pH in plasma efficacy was highlighted by Yamazaki et al. (2011) [16], who found significant bactericidal effects only at acidic pH (4.5), where ROS played a critical role in microbial killing. Koban et al. (2011) [55] demonstrated that volume dielectric barrier discharge (VDBD) plasma operated with argon gas achieved a 5.38 log_10_ CFU reduction in *S. mutans*, outperforming 0.1% chlorhexidine (CHX), which only achieved 3.36 log_10_. Liu et al. (2017) [26] extended these findings by demonstrating that LTP bacterial viability was reduced by ≥97% in single species biofilms of *S. mutans* and *S. sanguinis* when an atmospheric pressure non-equilibrium plasma jet operated with a helium-oxygen mixture was applied. Similarly, Yang et al. (2011) [17] achieved over 99% reduction in *S. mutans* in 13 s of argon-based LTP brush application.

Multispecies biofilms also exhibited strong susceptibility to LTP treatment. Koban et al. (2011) [55] reported that VDBD plasma reduced CFUs in saliva-derived biofilms by 5.67 log_10_, far surpassing the 1.50 log reduction observed with CHX, but the plasma device type and operating conditions critically influenced antimicrobial performance. The authors directly compared three plasma modalities—kINPen plasma jet, hollow dielectric barrier discharge (HDBD), and VDBD—finding that VDBD was the most effective in both mono- and multispecies biofilms. Nam et al. (2018) [30] evaluated the pre-treatment of extracted pre-molars with 15% carbamide peroxide (e.g., bleach) and dielectric barrier discharge (DBD) plasma operated with argon gas on *S. mutans* biofilm formation. The authors observed that plasma-assisted bleaching with 15% carbamide peroxide effectively reduced bacterial adhesion without causing detrimental surface changes to the enamel.

Puca et al. (2024) [52] applied direct air soft jet plasma that produces NO, NO_2_, and N_2_O to *S. mutans* biofilm and biofilms formed by a mixture of microorganisms isolated from the saliva of a patient with periodontitis, observing that the treatment for 60 s effectively reduced these biofilms. By analyzing multispecies biofilms, Figueira et al. (2024) [47] applied a plasma jet operated with argon gas (kINPen 09, Neoplas Med GmbH, Greifswald, Germany) for 30, 60, and 120 s to biofilms formed by *C. albicans*, *L. casei*, and *S. mutans*, observing significant reductions in viability and biofilm thickness following plasma exposure. Liu et al. (2017) [26] further support these observations, confirming that dual-species *S. mutans* + *S. sanguinis* biofilms experienced a ≥97% reduction in viability when treated with an atmospheric non-thermal argon/oxygen plasma brush for 2 min.

The synergistic effects of LTP with conventional antimicrobial agents were also evident. Koban et al. (2013) [15] found that LTP operated with argon gas (kINPen 09 Neoplas Med GmbH, Greifswald, Germany) combined with hydrogen peroxide (H_2_O_2_) or sodium hypochlorite (NaOCl) resulted in near-complete bacterial inactivation across *S. mutans*, saliva-derived, and subgingival biofilms. Koval’ovà et al. (2013) [19] investigated *Streptococci* biofilms using positive streamer corona and negative Trichel pulses, generated by high-voltage DC and pulsed power supplies. A positive streamer corona produces continuous, filamentary discharges that propagate outward due to strong local ionization. A negative corona generates intermittent Trichel pulses, which are rapid current spikes caused by space charge near a negatively biased electrode. The authors observed up to a 2.5 log_10_ reduction after 10 min of treatment and concluded that cold plasma generated from both DC positive streamer corona and negative Trichel pulses, as well as positive and negative pulsed corona, effectively reduced bacterial populations in *Streptococci* biofilm. Notably, spraying water during plasma exposure enhanced bacterial inactivation, achieving 3.3 log_10_ reductions within only two minutes, highlighting a powerful synergy between hydration and plasma-generated reactive species.

Imaging techniques such as confocal laser scanning microscopy (CLSM) and SEM were instrumental in verifying biofilm disruption. Figueira et al. (2024) [47] confirmed biofilm thinning and biomass reduction post-LTP treatment, while Puca et al. (2024) [52] demonstrated consistent microbial killing across varied field intensities. Nima et al. (2021) [39] and Huang et al. (2013) [20] provided mechanistic insights into oxidative damage, membrane lysis, and DNA fragmentation associated with plasma-induced microbial death.

Biofilm maturity and treatment duration also influenced LTP efficacy. Huang et al. (2013) [20] used a micro-plasma system operated with argon gas on 24-h *S. mutans* biofilms and observed progressive membrane damage over time. Although 300 s of plasma exposure was required for complete disinfection, bacterial structural deterioration was visible much earlier. These findings echo those of Yang et al. (2011) [17], who observed noticeable cell deformation and debris after just 5–15 s of plasma treatment (LTP brush that operated with argon gas) on *S. mutans.* Liu et al. (2017) [26] corroborate these results, as confocal laser scanning microscopy revealed predominantly dead bacteria in *S. mutans* biofilms following plasma treatment, while maintaining their chain-like structures.

Overall, LTP has consistently demonstrated strong antimicrobial efficacy against both mono- and multispecies biofilms, highlighting its potential in targeting cariogenic biofilms. Studies show that LTP effectively targets *S. mutans* biofilms, reducing bacterial viability and causing structural deformation, as seen through SEM and CLSM imaging. When combined with conventional antimicrobial agents like hydrogen peroxide and sodium hypochlorite, LTP enhances bacterial inactivation. Multispecies biofilms also showed significant reductions in bacterial counts and biofilm thickness, with LTP outperforming traditional disinfectants such as chlorhexidine. Factors such as device type, gas composition, and treatment conditions influenced its effectiveness. Overall, LTP’s versatility and synergy with other treatments make it a promising adjunctive tool for cariogenic biofilm management and a valuable addition to decontamination strategies.

### 4.2. LTP on Peri-Implantitis-Related Biofilms

The management of peri-implantitis, particularly through effective biofilm control, remains a significant clinical challenge. Complex microbial biofilms formed on titanium surfaces within peri-implant tissues are major contributors to persistent infection and inflammation, often complicating treatment outcomes. LTP has garnered increasing attention for its antimicrobial effects and its potential to enhance titanium surface decontamination in the treatment of peri-implantitis. LTP technology has emerged as a promising solution for biofilm removal, thanks to its ability to disrupt bacterial cells, modify surface properties, and enhance osseointegration, without damaging the titanium substrate.

Titanium, a commonly used material in dental implants, is particularly vulnerable to biofilm formation, which can lead to peri-implant infections and compromised osseointegration. Biofilm formation on the titanium surfaces of dental implants plays a crucial role in dental implant success, as it can lead to peri-implant infections and compromised osseointegration. Studies by Liu et al. (2011) [56] and Rupf et al. (2011) [61] demonstrated the effectiveness of LTP in reducing bacterial growth on rough titanium surfaces. Liu et al. (2011) [56] employed an atmospheric pressure non-equilibrium plasma jet with a helium-oxygen mixture, positioned at a 10 mm nozzle-to-surface distance, to treat biofilms of *Streptococcus sanguinis*, *Porphyromonas gingivalis*, and *Fusobacterium nucleatum* grown for 7 days on titanium discs. In a similar study, Rupf et al. (2011) [61] utilized a custom-built non-thermal plasma device with helium gas at a 2 mm nozzle-to-sample distance, targeting 3- to 5-day-old biofilms of *S. mutans*, *S. oralis*, and *P. gingivalis* on titanium surfaces. Both studies found that LTP treatment significantly reduced bacterial counts and biofilm mass, highlighting its potential as an effective method for biofilm management on titanium surfaces. Hui et al. (2021) [36] and Hui et al. (2021) [37] used a spark plasma pen jet at a 5 mm distance from the titanium surface to treat complex human biofilms obtained from patients with peri-implantitis grown for 96 h on titanium discs. Their findings confirmed that LTP treatment effectively reduced bacterial counts [36,37] without altering Ti surface topography [37]. Idlibi et al. (2013) [62] exposed titanium discs to in situ oral biofilm formation in five volunteers for 72 h and treated them with a microwave-based LTP source operated with an O_2_ admixture at a 2 mm distance, resulting in significant reductions in biofilm viability, biomass, and structural integrity, as confirmed by SEM. Preissner et al. (2016) [25] evaluated *Streptococcus mitis* biofilms cultured on microrough titanium dental implants for 84 h under anaerobic conditions, treated with an argon-based LTP source (Plasma ONE device, GmbH, Nievern, Germany) applied at an 8 mm tip-to-sample distance for 60 s or 120 s. They found that LTP significantly reduced CFU counts compared to the negative control. Fluorescence microscopy confirmed a higher proportion of dead cells in the LTP-treated groups, with no surface alterations observed. Ulu et al. (2018) [31] studied *S. aureus* biofilms grown for 7 days on titanium discs simulating dental implant surfaces. They applied LTP for 2 min at a fixed 1 mm discharge gap. The study showed that LTP demonstrated the highest level of biofilm inactivation, outperforming laser treatments, with no changes in surface roughness and a safe thermal profile maintained. Panariello et al. (2022 [46], 2024 [13]) investigated the effects of an argon-based LTP device (kINPen MED, Neoplas Med GmbH, Greifswald, Germany) on titanium surfaces to evaluate its impact on peri-implantitis-related biofilms, including *Actinomyces naeslundii*, *P. gingivalis*, *S. oralis*, and *Veillonella dispar*. Their findings demonstrated that LTP treatment significantly reduced bacterial counts [13,46] while maintaining the integrity of reconstituted oral epithelium in vitro [13], highlighting its potential as a safe and effective method for biofilm decontamination in the context of peri-implantitis. Matthes et al. (2022 [44], 2023 [45]) investigated the effects of LTP operated with a helium/oxygen gas mixture in combination with mechanical cleaning methods (such as water jets) on titanium surfaces, specifically on subgingival plaque biofilms, demonstrating superior biofilm removal and decontamination; observed that combined waterjet and LTP treatment effectively removed biofilms from titanium implants; and reduced inflammatory potential without inducing non-specific immune activation, suggesting potential for peri-implantitis treatment.

A key advantage of plasma treatment is its ability to modify the surface properties of titanium. Cavalcanti et al. (2014) [22] explored how surface modifications, specifically cold plasma nitridation of titanium, could influence biofilm composition. They applied ionic plasma containing air, noble gases, and nitrogen for 150 s to one face of titanium nitride (TiN) discs under high vacuum. Their study found that biofilms formed on TiN discs showed higher levels of *S. oralis* and *Fusobacterium nucleatum* compared to standard titanium, indicating that the surface properties of TiN influenced bacterial colonization, though total microorganism counts were similar between Ti and TiN surfaces. These findings suggest that surface modifications, such as cold plasma nitridation, may selectively affect the composition of the oral biofilm without increasing overall microbial load, adding a layer of complexity to plasma treatments on titanium. These changes can enhance the material’s hydrophilicity and surface energy, improving tissue integration and potentially enhancing osseointegration. Lee et al. (2019) [32] found that LTP treatments (plasma generated using compressed air gas at a 5 L/min flow rate) for 2 or 10 min increased surface hydrophilicity and energy without altering the topography of titanium discs, which is crucial for maintaining implant stability. The increased surface wettability was particularly beneficial in reducing bacterial adhesion, especially for gram-negative bacteria such as *Klebsiella* species, which showed significant membrane damage following plasma exposure. Additionally, Ji et al. (2023) [49] explored the use of LTP (argon + 1% O_2_) on anodized titanium surfaces, finding that plasma treatment significantly reduced *S. mutans* biofilm thickness while leaving the surface structure intact. Plasma treatment increased surface hydrophilicity by altering the chemical bonds on the titanium surface (e.g., increasing the O–H/TiO_2_ bonds), suggesting that surface modifications can play a key role in biofilm resistance and bacterial adhesion. Further supporting these findings, Matos et al. (2017) [27] evaluated the effects of surface modifications on titanium discs treated with glow discharge plasma (GDP) and plasma-enhanced chemical vapor deposition. The surfaces were then subjected to oxygen plasma treatment, and biofilm formation was assessed at early (16.5 h) and mature (64.5 h) stages, using a biofilm composed of *Streptococcus sanguinis*, *Actinomyces naeslundii*, and *Fusobacterium nucleatum*. The study found no significant differences in CFU/mL across the treated surfaces, including those exposed to GDP, compared to controls. This suggests that while plasma treatments may modify the surface properties of titanium, they do not promote bacterial colonization, indicating that plasma treatment does not contribute to enhanced biofilm formation.

Several recent studies have demonstrated the effectiveness and clinical relevance of LTP treatment using argon-based plasma sources for decontaminating titanium surfaces and treating biofilm-related infections. Carreiro et al. (2019) [11] utilized an argon-based LTP device (kINPen 09 Neoplas Med GmbH, Greifswald, Germany) to treat *P. gingivalis* biofilms cultured on titanium discs for 5 days. The plasma treatment, applied for 1 or 3 min, significantly reduced CFU/mL compared to negative and gas-only controls. Although slightly less effective than CHX, plasma treatment visibly reduced biofilm mass while preserving gingival epithelial cell viability, with minimal histological damage. Similarly, Haude et al. (2024) [51] explored the use of argon-based LTP on subgingival plaque biofilms from periodontitis patients. Plasma alone showed limited efficacy in biofilm removal, but when combined with glycine air-polishing, it achieved complete initial decontamination. Despite some regrowth of biofilms by day five, these findings highlight the potential of argon-based LTP-assisted cleaning as a powerful adjunctive treatment. In a study by Kamionka et al. (2022) [43], argon-based LTP was applied to *S. mutans* and *P. gingivalis* biofilms on both sandblasted and anodized titanium surfaces. Plasma treatment alone reduced fluorescence by 15.4–25.2%, but when combined with air-polishing using glycine or erythritol + CHX, significant reductions (77.9–92.5%) were observed, with effects lasting up to 5 days. The combination of LTP and mechanical cleaning provided sustained antimicrobial effects, particularly on sandblasted surfaces. Panariello et al. [13,46] demonstrated the efficacy of argon-based LTP against *A. naeslundii*, *P. gingivalis*, *S. oralis*, and *V. dispar*. Matthes et al. [44,45] expanded the application of argon-based LTP in combination with mechanical cleaning methods, such as water jets, to achieve superior subgingival plaque removal, reinforcing the integration of plasma technology with conventional therapies for enhanced clinical outcomes. Hong et al. (2022) [42] investigated the effects of pure argon and argon with oxygen LTP brushes on *P. gingivalis* biofilms formed over stainless-steel coupons for 3 or 5 days, demonstrating that LTP significantly reduced biofilm formation. Notably, the argon + 1% O_2_ treatment exhibited superior efficacy compared to pure argon.

Among the studies reviewed, only Canullo et al. (2023) [60] and Küçük et al. (2019) [59] conducted clinical trials, providing valuable insights into the real-world applications of LTP for managing peri-implantitis and periodontitis-related biofilms. Both utilized argon-based cold atmospheric plasmas. Canullo et al. performed a randomized controlled trial investigating the preventive potential of argon plasma pre-treatment on titanium healing abutments in vivo. The plasma-treated group demonstrated significantly reduced plaque accumulation and bleeding on probing compared to non-treated controls, with a microbial shift favoring early colonizers (e.g., *Streptococcus mitis*, *S. gordonii*) over pathogenic late colonizers like *Neisseria oralis* and *Parvimonas micra*. However, no significant effect on the peri-implant soft tissue phenotype was observed. This suggests that while LTP may not alter tissue architecture, it can effectively shape the initial biofilm toward a more commensal profile. In contrast, Küçük et al. investigated the use of LTP as an adjunct to non-surgical periodontal therapy in patients with periodontitis. In this split-mouth, randomized trial, patients receiving LTP treatment after scaling and root planing showed greater clinical attachment level gains, significant reductions in inflammation, and more effective suppression of red complex bacteria (*P. gingivalis*, *Tannerella forsythia*, and *Treponema denticola)* compared to controls. Importantly, LTP appeared to delay bacterial recolonization, contributing to longer-term microbial stability and improved clinical outcomes. While Canullo [60] focused on preventing peri-implant biofilm formation and inflammation, Küçük [59] emphasized adjunctive disinfection and healing in active periodontal pockets. Together, these clinical trials underscore the multifaceted potential of LTP in both peri-implant and periodontal therapy, whether as a preventive surface modification strategy or as a therapeutic adjunct in active disease management.

These studies collectively underscore the potential of LTP to improve biofilm decontamination on titanium surfaces in peri-implantitis treatment. The various types of LTP devices employed across studies demonstrate the versatility of this technology in effectively targeting microbial biofilms of different ages and compositions. Although results vary slightly depending on the treatment conditions, LTP consistently shows promise in reducing the biofilm load, enhancing surface properties, and maintaining biocompatibility, offering a viable adjunctive treatment for dental implant-related infections.

### 4.3. LTP on Endodontic Biofilms

In evaluating the antimicrobial efficacy of LTP in root canal disinfection, *E. faecalis* emerges as the primary bacterial species investigated across most studies.

The studies by Pan et al. (2013) [18] and Li et al. (2015) [23] show that LTP can achieve significant reductions in *E. faecalis* CFUs within root canals, with Pan et al. [18] utilizing a non-thermal atmospheric pressure plasma jet operated with a mixture of argon and oxygen, demonstrating complete bacterial elimination after 10 min of plasma exposure. Li et al. (2015) [23] utilized a similar plasma and corroborate this finding, showing that cold plasma exposure led to a time-dependent bactericidal effect, achieving complete biofilm inactivation at 12 min. In contrast, Schaudinn et al. (2013) [58] used an LTP operated with He/O_2_ and found LTP’s efficacy to be somewhat less than that of 6% sodium hypochlorite (NaOCl), despite demonstrating biofilm reduction in root canals. This suggests that plasma’s efficacy may be enhanced by optimizing treatment parameters, such as exposure time, which varies among studies.

While plasma treatment has been shown to effectively reduce biofilm biomass, it is not without limitations compared to traditional endodontic treatments like NaOCl and calcium hydroxide (Ca(OH)_2_). For example, Ballout et al. (2018) [28] reported that NaOCl combined with passive ultrasonic irrigation (PUI) outperformed all LTP treatments (kINPen MED, Neoplas Med GmbH, Greifswald, Germany) in biofilm eradication. The superior performance of NaOCl + PUI is attributed to its enhanced ability to penetrate the complex anatomy of root canals, a capability that was not fully achieved by the plasma treatments tested in the same study. Similarly, Asnaashari et al. (2022) [41] found that the bacterial elimination achieved by triple antibiotic paste was far superior to that of LTP (helium/oxygen plasma jet), underscoring the ongoing effectiveness of antibiotic-based treatments in biofilm management. The results from these studies indicate that while plasma treatments offer a promising alternative, they still fall short when compared to the gold standard of NaOCl in terms of biofilm eradication from root canals.

A striking observation across several studies is the variability in plasma treatment protocols, including plasma gas composition, exposure time, and power. For instance, Li et al. [23] employed a cold plasma jet with a gas flow rate of 5 L/min and a continuous exposure duration of up to 12 min, using an argon/oxygen mixture. This setup was highly effective in biofilm inactivation, with SEM and CLSM analyses showing complete bacterial detachment and rupture. In contrast, Schaudinn et al. [58] utilized a DBD system with a lower gas flow rate of 1 L/min, delivering intermittent exposure in three 10-min sessions. The helium/oxygen mixture used in their study, combined with the lower flow rate and pauses between exposures, resulted in a less aggressive treatment, which led to only partial biofilm reduction. Longer treatment durations generally correlated with better outcomes, as seen in both Pan et al. [18] and Li et al. [23], where longer exposures led to greater bacterial reductions. This was supported by the findings of Theinkom et al. (2019) [33], where a 10-min cold atmospheric plasma treatment resulted in a ≥5 log_10_ CFU reduction, demonstrating the importance of optimizing exposure times to achieve clinically relevant disinfection.

The mechanism by which plasma inactivates bacterial biofilms appears to involve multiple factors, including reactive oxygen species (ROS), UV radiation, and charged particles, as discussed by Pan et al. [18] and Li et al. [23]. SEM and CSLM imaging from these studies revealed biofilm disruption and bacterial rupture post-treatment, suggesting that plasma’s bactericidal effects are primarily due to oxidative damage and UV-induced DNA damage. In addition, Takahashi et al. (2015) [24] highlighted the role of reactive species such as oxygen and hydroxyl radicals in biofilm inactivation, emphasizing the importance of these reactive species in expanding the area of biofilm inactivation beyond the direct irradiation spot. This ability to treat a larger area is particularly relevant in clinical settings, where biofilm structures are often more extensive and complex.

Mechanical safety is another critical factor in evaluating the feasibility of plasma treatments for clinical use. Several studies, such as those by Li et al. [23] and Theinkom et al. (2019) [33], reported no significant changes in dentin microhardness or surface roughness after plasma exposure, which is a favorable finding when considering the safety of plasma treatments in clinical practice. This is particularly relevant when comparing plasma treatments to NaOCl, which can potentially alter dentin properties and lead to surface corrosion. Zarif et al. (2021) [40] also demonstrated that the combination of plasma with fluoride could enhance antibacterial effects without compromising the structural integrity of dentin, suggesting a synergistic effect that may improve clinical outcomes in the long term.

Although plasma treatment shows significant promise, the variability in efficacy, treatment protocols, and comparison to traditional methods suggests that further optimization is needed before plasma can become a routine clinical practice. As noted by Asnaashari et al. [41], the in vitro models used in these studies, such as single-species biofilms, limit the applicability of these findings to real-world clinical situations, where polymicrobial biofilms are often present. Moreover, studies like Zhu et al. (2023) [50] emphasize the need for additional in vivo investigations to validate the effectiveness of plasma treatments in living tissues.

Collectively, the findings underscore the potential of plasma-based therapies as a promising alternative to conventional endodontic disinfection methods. Although plasma treatment consistently demonstrates a reduction in biofilm viability, discrepancies in experimental designs, plasma parameters, and benchmarking against traditional techniques indicate the need for further standardized research to fully assess its clinical applicability.

While LTPs offer a novel approach to biofilm eradication in endodontics, they currently do not surpass traditional methods such as NaOCl in terms of efficacy. Plasmas’ advantages lie in their ability to deliver targeted antimicrobial effects without direct contact, minimizing the risk of damage to surrounding tissues. However, continued research into optimizing treatment parameters, understanding the full range of plasma’s mechanisms of action, and exploring combination therapies with other disinfection modalities will be crucial to translating these promising results into clinically viable treatments.

### 4.4. LTP on Biofilms Associated with Oral Candidiasis and Antibiotic-Resistant Strains

LTP has emerged as a promising technology for the inactivation of biofilms, including those formed by *Candida* species, especially *C. albicans*, a common fungal pathogen responsible for oral candidiasis. The ability of LTP to effectively target and disrupt biofilms, including those involving antibiotic-resistant strains, such as *Staphylococcus aureus*, presents an exciting opportunity in the development of alternative antimicrobial treatments. Several studies have provided insight into the antimicrobial efficacy of LTP on *C. albicans* biofilms and antibiotic-resistant biofilms, shedding light on its potential application in clinical settings.

Delben et al. (2016) [12] demonstrated that LTP treatment using an argon-based plasma (kINPen 09 Neoplas Med GmbH, Greifswald, Germany) could significantly reduce viable counts of *C. albicans* biofilms. In their study, both single-species and dual-species biofilms (including *S. aureus*) were exposed to LTP, and the results indicated a substantial decrease in the viability of *C. albicans* and *S. aureus* cells. The reduction in biofilm viability was confirmed through various techniques such as CLSM and SEM, which revealed the disruption of cell walls and membrane rupture following plasma treatment. Furthermore, fluorescence microscopy confirmed the presence of ROS in the LTP-treated biofilms, which play a critical role in plasma-induced microbial inactivation. This study highlights the potential of LTP as an effective alternative to traditional antifungal agents like fluconazole, particularly in biofilm-related infections, which are notoriously resistant to standard therapies.

Nagay et al. (2020) [34] also investigated the efficacy of LTP treatments (sulfur hexafluoride-based LTP, hexamethyldisiloxane + argon-based LTP, and oxygen-based LTP) on biofilms of *C. albicans* and *S. oralis*. Their study revealed that LTP treatments induced surface modifications on the resinous denture liner, which contributed to enhanced microbial inhibition. SEM images showed alterations to the surface topography of the liner after LTP exposure, which were associated with a reduction in *C. albicans* colonization. Importantly, these surface changes did not cause significant damage to the underlying material, highlighting LTP’s ability to inhibit fungal growth while preserving the integrity of dental materials like acrylic resin. These findings are consistent with those of Delben et al. (2016) [12], further supporting the potential of plasma-based technologies in managing candidal biofilms.

Leite et al. (2021) [38] extended this line of investigation by assessing the effects of LTP (helium DBD jet) and antifungal agents on *C. albicans* biofilms. They found that LTP alone, especially when applied for 5 min, was more effective than the polyene antifungals nystatin and amphotericin B in reducing biofilm viability. While no synergy was observed between LTP and antifungal agents, the study emphasized that LTP could be a highly effective standalone treatment, particularly in cases where antifungal resistance is a concern. The absence of synergy may reflect biofilm protective responses, such as increased exopolymer matrix production, which can mitigate the effects of both oxidative stress from LTP and the antimicrobial action of antifungals.

Avukat et al. (2023) [48] focused on the use of helium plasma for reducing *C. albicans* biofilm formation on polished polymethyl methacrylate (PMMA) surfaces. The study showed that helium plasma, particularly at higher concentrations (100% helium), significantly reduced both *C. albicans* viability and biofilm formation, as confirmed by MTT and crystal violet assays. SEM images supported these findings, revealing a marked reduction in *C. albicans* adhesion and biofilm structure on plasma-treated surfaces. Interestingly, surface roughness was not significantly altered by the plasma treatment, suggesting that the reduction in biofilm formation was due to changes in surface chemistry, such as increased hydrophilicity and surface energy, rather than physical alterations. These findings underscore the versatility of helium plasma as a treatment option, particularly for materials like PMMA, commonly used in dentures, where fungal contamination can lead to denture stomatitis.

Morais et al. (2025) [54] provided further evidence for the efficacy of LTP (helium DBD jet) in reducing *C. albicans* biofilms on titanium alloy surfaces, which are commonly used in implant-supported dentures. After LTP treatment, there was a significant reduction in *C. albicans* CFUs and a decrease in pseudohyphae formation, indicating the effectiveness of LTP in disrupting biofilm architecture. SEM images confirmed that plasma treatment led to a reduction in yeast aggregates on the titanium surface, which is crucial in preventing infection on implant materials. This study aligns with the growing body of evidence suggesting that LTP can be an effective method for reducing fungal contamination on medical and dental implant surfaces, offering a potential alternative to conventional antifungal treatments.

On the other hand, Hafner et al. (2018) [29] studied the bactericidal efficacy of LTP (Plasma ONE device, GmbH, Nievern, Germany) in comparison to established antimicrobial therapies such as antimicrobial photodynamic therapy. Their study focused on biofilms formed by *Acinetobacter baumannii* and *S. aureus*, both of which are well-known antibiotic-resistant strains, commonly found in clinical settings like dental or oral infections. They observed that while LTP showed promise in its ability to generate reactive ROS and RNS for antimicrobial activity, its effectiveness was significantly reduced when the water film over the bacterial culture exceeded 0.3 mm in thickness. This limitation is particularly relevant for moist environments like the oral cavity, where the depth of water or tissue may impair LTP’s penetration and efficacy. This finding underscores a challenge in applying LTP to oral candidiasis, where biofilm formation on moist surfaces could limit the depth of plasma penetration. While LTP has shown effectiveness in reducing biofilm viability on dental materials in vitro, as demonstrated by Delben et al. [12], Nagay et al. [34], Morais et al. [54], Avukat et al. [48], and others, future studies may need to focus on optimizing plasma parameters or exploring devices with higher output to improve efficacy in moist or deeper biofilm environments.

These studies highlight the promising potential of LTP in treating *C. albicans* biofilms on dental materials and implants. LTP generates reactive oxygen and nitrogen species (RONS), which cause oxidative stress, damage cell walls and membranes, and lead to microbial death. LTP’s versatility in treating biofilms on various surfaces—such as denture liners, titanium alloys, and PMMA—supports its clinical application for oral candidiasis. However, challenges remain, including penetration through a thicker biofilm matrix. Maximizing LTP’s efficacy requires further optimization of plasma parameters, exploration of combination therapies, and in vivo studies. Therefore, LTP offers a promising alternative for treating *C. albicans* biofilms and antibiotic-resistant strains, but continued research is essential for translating these findings into effective clinical treatments.

### 4.5. Influence of the Plasma Source and Discharge Mechanism on Antimicrobial Outcomes

The varying antimicrobial responses observed across studies can be partially attributed to fundamental differences in plasma generation methods, discharge modes, and gas-phase chemistry among the LTP devices used. For example, volume dielectric barrier discharge (VDBD) systems produce diffuse, large-area discharges through capacitive coupling across dielectric layers, enabling broad and uniform treatment coverage, likely contributing to their superior efficacy in both mono- and multispecies biofilms as shown by Koban et al. (2011) [55]. In contrast, plasma jets like the kINPen, powered by argon or helium-oxygen mixtures, create narrow, focused plasma plumes with high densities of reactive oxygen and nitrogen species (RONS) [9,10]. While these jets offer precise control and high reactivity, their spatially confined output may limit coverage unless scanned across larger surfaces.

Positive streamer corona discharges, generated by applying high-voltage DC to sharp electrodes, form rapidly propagating ionization fronts with continuous RONS production, favoring broader oxidative stress. Conversely, negative corona discharges produce Trichel pulses—high-frequency, localized current bursts that create transient and spatially confined plasmas [63], potentially resulting in less homogeneous biofilm exposure. Despite these limitations, both corona modes were effective at reducing *Streptococcal* biofilms, especially when paired with hydration to enhance radical penetration, as observed in the study by Koval’ová et al. [19].

Similarly, the argon plasma brush and soft jet air plasma systems used by Yang et al. (2011) [17] and Puca et al. (2024) [52] delivered strong antimicrobial effects, albeit through differing mechanisms. The plasma brush operates with high-density, low-temperature discharge directly at the target site, whereas the soft jet delivers nitrogen oxides (NO, NO_2_, N_2_O) via air plasmas, leveraging reactive nitrogen species for microbial killing. These differences suggest that both discharge structure (streamer vs. diffuse vs. pulsed) and gas composition (e.g., Ar, He/O_2_, air) critically influence the type and abundance of reactive species generated, spatial plasma reach, and ultimately, biofilm inactivation efficiency.

Studies investigating LTP applications in peri-implantitis-related biofilms reveal similar dependencies on plasma source and discharge characteristics. Argon-based plasma jets such as the kINPen MED (Panariello et al. [13,46], Carreiro et al. [11], Kamionka et al. [43]) generate stable, narrow plumes rich in RONS, enabling effective localized biofilm eradication while preserving titanium surface integrity and epithelial viability. Spark plasma pens (Hui et al. [36,37]), by contrast, employ pulsed discharges that generate transient, high-energy reactive species bursts, offering deep biofilm penetration but with potentially less uniform exposure due to their point-source nature.

Microwave-driven plasma sources (Idlibi et al. [62]) and compressed air plasma jets (Lee et al. [32]) differ in their energy coupling mechanisms and reactive species profiles, with oxygen admixtures consistently enhancing antimicrobial efficacy via elevated ROS production. Glow discharge plasma (GDP) and plasma-enhanced chemical vapor deposition techniques (Matos et al. [27]) primarily induce surface chemical and wettability modifications rather than direct microbial killing, highlighting their utility in biofilm prevention or implant surface conditioning rather than active decontamination.

Furthermore, helium-oxygen plasmas (Liu et al. [56], Matthes et al. [44,45]) balance high reactivity and gentle surface interaction, effectively reducing biofilm load while maintaining titanium surface topography. Notably, combined treatment strategies integrating LTP with mechanical biofilm disruption—such as glycine air-polishing or water jets (Haude et al. [51], Matthes et al. [44,45], Kamionka et al. [43])—yield superior clinical outcomes, suggesting synergistic benefits from physical removal and plasma-induced microbial inactivation.

These differences in plasma source design, discharge mode, and gas chemistry significantly influence the nature and concentration of reactive species generated, biofilm penetration depth, and titanium surface effects. Consequently, tailoring plasma parameters to the target biofilm composition and clinical scenario is critical. For example, argon-based plasmas with low-temperature, high-RONS jets are well suited for precise peri-implant biofilm decontamination without altering implant surfaces, while plasma modalities generating pulsed or volumetric discharges may better address mature or complex biofilms through enhanced penetration and reactive species flux.

Together, these findings underscore that plasma source configuration, discharge mechanism, and gas-phase chemistry are pivotal determinants of antimicrobial efficacy and surface compatibility in both dental caries and peri-implantitis contexts, guiding the optimization of LTP therapies for improved clinical outcomes.

### 4.6. Clinical Applications and Future Directions

While the evidence presented highlights the broad potential of low-temperature plasma (LTP) for oral biofilm management, it is essential to consider the practical implications for clinical dentistry. LTP has demonstrated effectiveness in managing oral biofilms related to conditions such as dental caries, peri-implantitis, endodontic infections, and oral candidiasis. This makes it a promising adjunct to traditional treatments, offering advantages such as minimal thermal damage, broad-spectrum antimicrobial action, and reduced risk of resistance development. However, further research is needed to standardize treatment protocols, including factors like the distance and time of application, which should be adjusted according to the type of LTP and gas used. Additionally, randomized clinical trials (RCTs) are essential to assess the long-term outcomes and real-world effectiveness of LTP in clinical settings. While promising results from in vitro, ex vivo, and in situ studies suggest that LTP could soon become an invaluable tool in the dental clinician’s arsenal for managing biofilm-related infections, a better understanding of its optimal application settings, including plasma parameters and treatment duration, will be crucial to enhancing its effectiveness and safety in clinical practice.

### 4.7. Strengths and Limitations

A major strength of this scoping review lies in the inclusion of 51 peer-reviewed studies, offering a broad and detailed overview of the current evidence surrounding the effects of LTP on oral biofilms. This extensive coverage enables the identification of key research trends, recurring methodological approaches, and emerging areas of interest. The volume and diversity of the included literature enhances the robustness of the thematic synthesis and provides a strong foundation for highlighting knowledge gaps and shaping future research directions. The review contributes meaningful insights into the complex interplay between LTP technologies and biofilm behavior in the oral environment.

However, several limitations should be acknowledged. Considerable heterogeneity in plasma parameters and types, biofilm models, treatment protocols, and experimental conditions restricts the ability to draw direct comparisons or perform meaningful cross-study analyses. The exclusion of non-English publications and the grey literature may introduce publication bias and reduce the comprehensiveness of the review. Furthermore, the absence of a formal quality assessment limits the capacity to evaluate the methodological rigor of the studies included. The rapid pace of technological advancement in this field also poses challenges in ensuring the ongoing relevance of the findings. Inconsistent terminology across studies and reliance on reviewer interpretation during data extraction may introduce additional subjectivity and bias. As such, while the findings offer valuable direction, they should be interpreted with appropriate caution.

## 5. Conclusions

LTP has demonstrated significant potential as an effective antimicrobial strategy for managing oral biofilms. Its antimicrobial action primarily arises from the generation of reactive oxygen and nitrogen species (RONSs), UV radiation, and charged particles, which together disrupt microbial membranes, damage intracellular components, and degrade biofilm matrices. This multifaceted mode of action enables effective microbial inactivation while maintaining favorable biocompatibility. Beyond its direct antimicrobial efficacy, LTP has shown promise in enhancing surface properties, particularly in clinical contexts such as peri-implantitis, where biofilm control on implant surfaces is critical. By increasing surface hydrophilicity and energy, LTP promotes improved tissue integration and implant stability. Moreover, LTP has demonstrated synergistic potential with conventional antimicrobial agents, amplifying therapeutic outcomes and positioning it as a potent adjunct in biofilm-associated oral infections.

However, the effectiveness of LTP depends on multiple factors, including treatment parameters (exposure time, distance), plasma device configurations (discharge type, gas composition), and the complex composition of target biofilms. These variables influence the production and spatial distribution of reactive species, thermal effects, and plasma coverage, all of which critically impact microbial inactivation efficiency and host tissue responses. To fully realize its full clinical potential, further research is needed to refine plasma delivery techniques, optimize device parameters, and elucidate in vivo mechanisms of action, including host tissue interactions and long-term safety.

While LTP represents a compelling alternative to traditional antimicrobial treatments like antibiotics and chlorhexidine, challenges remain in standardizing protocols and integrating LTP into existing clinical workflows. With continued technological advances and focused research efforts, LTP is well-positioned to emerge as a versatile and valuable tool for managing oral biofilm-related diseases, offering effective antimicrobial action with a reduced risk of resistance development.

## Data Availability

No new data were created or analyzed in this study. Data sharing is not applicable to this article.

## Supplementary Materials

The following supporting information can be downloaded at https://www.mdpi.com/article/10.3390/medsci13020079/s1, Table S1: Full search strategy for each electronic database; Table S2: Comprehensive summary of included studies detailing study design, funding, biofilm characteristics, type of LTP used and application details, main outcomes, and conclusions; Table S3: List of excluded studies and reasons for exclusion.
