# Supplementary material for: Exploring the Efficacy of Low-Temperature Plasmas on Oral Biofilms: A Scoping Review
Source: Med Sci (Basel). 2025 Jun 18;13(2):79. doi: 10.3390/medsci13020079 (PMC12195150; doi:10.3390/medsci13020079)
Supplement: Supplementary file 1 [file medsci-13-00079-s001.zip › medsci-3642734-supplementary.pdf]

Table S1: Full search strategy for each electronic database.

**PubMed/MEDLINE:** ("Plasma Gases"[MeSH Terms] OR "cold plasma" OR "non-thermal plasma" OR "low temperature plasma") AND ("Biofilms"[MeSH Terms] OR "oral biofilm" OR "dental plaque" OR "microbial biofilm") AND ("Mouth"[MeSH Terms] OR "oral cavity" OR "dentistry" OR "oral health" OR "oral tissues") NOT (review[Publication Type])<br>Filter for English language only.
**Google Scholar:** ("cold plasma" OR "non-thermal plasma" OR "low temperature plasma") AND ("oral biofilm" OR "dental plaque" OR "microbial biofilm") AND ("oral cavity" OR dentistry OR "oral health" OR "oral tissues") -"review" -"book" -"poster" -"e-poster"<br>Filter for English language only and exclude citations.
**EBSCO**: (cold atmospheric plasma or cold plasma or low-temperature plasma) AND (oral biofilm or dental plaque or microbial biofilm) AND (oral cavity OR dentistry OR oral health OR oral tissues)

Table S2: Comprehensive summary of included studies detailing study design, funding, biofilm characteristics, type of LTP used and application details, main outcomes, and conclusions.

**Authors/ Year**	**Type of study**	**Funding**	**Characteristics of the biofilms**	**Type of LTP and application details**	**Main results**	**Conclusions**
Koban et al. (2011)	*In vitro and ex vivo*	German Ministry of Education and Research (BMBF, grant no. 13N9779).	*S. mutans* biofilms were grown on titanium discs, while saliva biofilms were formed using pooled saliva from healthy donors.	kINPen09 (argon gas [Ar]), hollow dielectric barrier discharge (HDBD) Ar + 11% O_2), and volume dielectric barrier discharge (VDBD) (Ar); exposure times: 1, 2, 5, and 10 min; 7 mm nozzle-to-biofilm distance.	Plasma was more effective than CHX against biofilms. CHX reduced CFUs by 3.36 for *S. mutans* and 1.50 for saliva, while VDBD Ar plasma reduced CFUs by 5.38 for *S. mutans* and 5.67 for saliva biofilm.	LTP treatment of dental biofilms on titanium discs was more effective than CHX, suggesting potential for peri-implant mucositis treatment.
Liu et al. (2011)	*In vitro and in vivo*	National Natural Science Foundation of China (No. 10875048).	*In vitro: P. gingivalis* biofilms cultured for 15 days onto cover slips in 24-well plates; LTP treatment applied after maturation (≥10 days). *In vivo:* LTP applied to rabbit oral mucosa; tissues examined at 1- and 5-days post-treatment.	Atmospheric pressure non-equilibrium plasma jet operated with a helium-oxygen (He+ O_2) mixture applied an 8 kV pulsed DC voltage with a 10 kHz frequency and 1600 ns pulse width. The nozzle-to-surface distance was 10 mm.	LTP effectively killed *P. gingivalis* biofilms without harming the surrounding tissue. In vivo, the plasma treatment was applied to the oral mucosa of rabbits, with no signs of tissue damage or irritation observed at 1- and 5-days post-treatment.	LTP effectively kill bacteria in biofilms without harming rabbit oral mucosa, based on clinical and pathological findings.
Yamazaki et al. (2011)	*In vitro*	Science Foundation of China (No. 10875048).	*S. mutans* biofilms were grown on 96-well plates, while *C. albicans and E. faecalis* were cultured in suspension.	Low-frequency plasma sterilization (Yoshida Dental Mfg. Co., Tokyo, Japan) with He, plasma jet 20 mm above surface, and plume diameter of 1 mm, applied for 2, 3, and 5 minutes.	The plasma jet significantly reduced biofilm microbes at pH 4.5.	LTP had a sterilizing effect on oral pathogenic microorganisms present in both the solid and liquid phases.
Yang et al. (2011)	*In vitro*	National Science Foundation (US NSF-CBET-0730505) and US National Institute of Health (1R43DE019041-01A1).	*S. mutans* and *L. acidophilus* biofilms grew in porous filter papers (to mimic tooth fissures), glass slides (smooth surfaces), and PTFE films (polymer surfaces) for 24 hours.	LTP brush that operates with Ar at low power (5–15 W) and produced gas temperatures between 30–65°C was applied to the biofilms fro 0 to 300s (2000sccm Ar flow rate, 10 w input power).	LTP Argon Brush effectively disinfects *S. mutans* and *L. acidophilus*, achieving over 99% reduction of *S. mutans* in 13 s.	LTP is effective in deactivating oral bacteria and could be a promising technique in various dental clinical applications.

Rupf et al. (2011)	*In situ*	US National Institute of Health (NIH) with grant number of 1R43DE019041-01A1. The	*In situ* 24 or 72 h oral biofilms grew on titanium discs mounted on custom splints worn by two male volunteers (31 & 43 yrs).	Custom-built non-thermal plasma (Leibniz Institute, Germany) with He, 2 mm nozzle-to-titanium sample distance, 2.0 l/min helium flow, 5 ms microwave pulse at 250 W, 3 or 5 W jet power, <0.5 mm width, 5 mm length.	Plasma treatment (3–5 W) raised surface temperature to max. ~43°C; no microbial growth was found on 24 h biofilms post-treatment. Partial reduction in 72 h biofilms was improved by air/water spray. Complete removal was achieved after a second plasma cycle (3 or 5 W), with no surface damage. Control samples showed viable biofilms and microbial growth.	LTP shows strong potential for antimicrobial use in dentistry, particularly for disinfecting biofilm-contaminated microstructured titanium surfaces.
Idlibi et al. (2013)	*In situ*	Saarland University (grant T6031500-09).	*In situ* oral biofilms grew on titanium discs exposed to the oral cavities of five healthy human volunteers for 72 hours.	Microwave-based LTP source (Leibniz Institute) operated with O_2 admixtures, delivering 5 µs pulses at 250W with variable mean power (3–5 W), and a fixed 2 mm working distance between the nozzle and sample surface.	LTP significantly reduced biofilm viability, biomass, and altered morphology. Scanning electron microscopy showed degraded biofilm structure and fewer surface bacteria. The efficacy of treatment correlated with the treatment duration and plasma power.	No single method achieved complete biofilm removal; however, LTP may provide effective support to established decontamination techniques for treatment of peri-implant diseases.
Koban et al. (2013)	*In vitro*	German Ministry of Education and Research (BMBF, Grant no. 13N9779).	*S. mutans* and multispecies saliva biofilm in titanium discs.	kINPen 09 (Ar). Titanium discs with biofilms were pretreated for 30 minutes with 0.1% CHX, 0.1% OCT, 0.1% PHMB, 0.1%, 0.6% NaOCl, 1.5% H_2O_2, and 20% EDTA, followed by 1-minute plasma treatment at 7 mm distance.	In *S. mutans* biofilms, plasma with hydrogen peroxide (H_2O_2) or sodium hypochlorite (NaOCl) eliminated bacteria; in saliva biofilms, NaOCl + plasma showed the highest reduction; and in subgingival biofilms, plasma with H_2O_2 achieved the strongest effect, with plasma enhancing most antiseptics across all models.	LTP significantly enhances the antimicrobial effects of common antiseptics across S. mutans, saliva-derived, and subgingival biofilms, with the strongest biofilm reductions achieved in combination with hydrogen peroxide and sodium hypochlorite.
Pan et al. (2013)	*In vitro*	MST Program of International Science and Technology Cooperation (grant no. 2009DFB30370); National Basic Research Program (grant no. 2007CB935602); Electro-Energetic Physics Program of the United States Air Force Office of Scientific Research (grant no. FA9550-08-1-0332).	*E. faecalis* biofilms on root canal sample.	Nonthermal atmospheric pressure plasma jet operated with Ar/O_2 (2%) applied for up to 10 minutes. The jet (5 cm) operated at 18 kV, 10 kHz, with a 5 L/min gas flow. Treatment was performed at ~5 mm distance, with gas temperatures between 25–31°C.	LTP significantly reduced *E. faecalis* in root canals, outperforming calcium hydroxide at 8–10 min and fully eliminating bacteria at 10 min; SEM and CSLM confirmed biofilm disruption and bacterial death.	LTP effectively inactivated *E. faecalis* biofilms in root canals, likely due to a combination of UV radiation, reactive species, and charged particles disrupting bacterial cells and biofilm structure

Schaudinn et al. (2013)	*Ex vivo*	Research grant (R21-DE020167-01A1) from the National Institute of Dental and Craniofacial Research (NIDCR).	Biofilms in root canals of extracted teeth.	Root canals were treated with LTP for 30 minutes (3 sessions of 10 minutes with 2-minute pauses) using a 6.5 kV plasma device. The device operated with a laminar gas flow (1 l/min) of a He/O_2 mixture (99:1), with the average power maintained below 0.5 W.	LTP groups showed significant biofilm reduction compared to saline solution, but not as effective as 6% NaOCl. LTP-treated samples displayed areas with both dead and live bacteria.	LTP displayed antimicrobial activity against endodontic biofilms in root canals but was not as effective as the use of 6 % NaOCl.
Koval'ová et al. (2103)	*In vitro*	Slovak Grant Agency VEGA 1/ 0668/11, Slovak Research and Development Agency APVV SK-CZ-0179-09 and SK-FR-0038-09.	Biofilms were formed from the Viridans group of *Streptococcus* obtained from the oral microflora of three volunteers (22-year-old woman and two 23-year-old men). The biofilms were cultivated on plastic surfaces for 24 hours.	Positive streamer corona and negative Trichel pulses, generated by high-voltage DC and pulsed power supplies, produced current pulses (10–20 kHz for positive, up to 1 MHz for negative) with amplitudes up to 150 mA. Plasma was applied 5 mm from the biofilms for 2, 5, and 10 minutes.	*Streptococci* biofilms were exposed to positive and negative DC corona discharges for 2, 5, and 10 minutes, achieving up to 2.5 log reduction. With water spraying, log10 reduction increased from 0.95 to 3.3 logs, with slightly better efficiency at 0.01 mL/min for positive corona.	The authors concluded that cold plasma from both DC positive streamer corona and negative Trichel pulses, as well as positive and negative pulsed corona, effectively reduced bacterial populations in *Streptococci* biofilm.
Huang et al. (2013)	*In vitro*	National Science Council of Taiwan (NSC 99 – 2221-E-006 – 013-MY3) and Medical Device Innovation Center (D100 – 21001).	*S. mutans* cultivated on glass substrates for 24 and 48 hours.	Micro-plasma system (ENI1 ACG-3B, MKS Instruments Inc., Rochester, NY, USA) with Ar, 23 W power, and radio-frequency ignition. Working distance: 3 mm.biofilms were treated at different time intervals (60, 180, 300 seconds).	Micro-plasma treatment of *S. mutans* biofilms caused significant bacterial damage, indicated by fluorescence shift and SEM images. Bacterial survival decreased with treatment time, achieving complete disinfection in 300 seconds for 24-hour biofilms.	Argon micro-plasma effectively disinfected *S. mutans* biofilm, significantly damaging the bacteria. Disinfection efficiency increased with shorter distances and thinner biofilms.
Blumhagen et al. (2014)	*In vitro*	NIH (5R01DE021431 and 5R44DE019041).	*L. acidophilus* and *S. mutans* biofilms grown on hydroxyapatite disks for 24 hours.	Argon LTP brush (3 W, 0.5 kV, 6 mA, 3000 sccm argon flow, with optional oxygen admixture) applied at ~5 mm distance on biofilms. Plasma was applied in 20-second intervals, covering approximately 1/6 of the sample surface at a time.	Plasma inactivated *L. acidophilus* and *S. mutans* in 6–13 s at low/medium inoculum levels; high concentrations showed ~2.5 log reduction. SEM showed surface cell damage, with deeper layers intact. Inactivation was due to plasma, not heat.	DC atmospheric plasma effectively deactivated oral bacteria on an enamel analogue, destroying the top bacterial layer while leaving deeper cells intact. SEM confirmed that cell debris shielded underlying bacteria.
Cavalcanti et al. (2014)	*In vitro*	Sao Paulo Research Foundation - FAPESP (Grants 2011/03242-0 and 2010/ 07894-9).	A multispecieis biofilm containing *A. naeslundii, V. dispar, F. nucleatum, S. mutans, S. oralis, C. albicans* grew for 64.5 h on titanium (Ti) and cold plasma-nitrided titanium (TiN) discs.	Ionic plasma containing air, noble gases, and nitrogen was applied for 150 s to one face of the TiN discs under high vacuum. The machine was fabricated by the Physical Institute of University of Campinas (UNICAMP).	The SEM images and 3D reconstructions of the CLSM images revealed similar biofilm structures on both Ti and TiN discs. The viable cell counts showed higher levels of *S. oralis* and *F. nucleatum* on TiN surfaces, while the total microorganism count was similar between the two groups.	The findings suggest that the surface properties of TiN may influence the composition of the oral biofilm, particularly affecting the colonization of specific bacterial species.
Li et al. (2015)	*In vitro*	985 Program of Peking University	*E. faecalis* (ATCC 29212) biofilms grown for 3 weeks in root canals of 100 caries-free, single-rooted human teeth, incubated in BHI (Brain-heart infusion agar) broth at 37°C, refreshed every other day.	Non-thermal atmospheric pressure plasma jet with Ar (98%)/O_2 (2%) admixture, 5 L/min gas flow, 10 mm working distance, applied for 3, 6, 9, or 12 minutes; mean plasma temperature ~30.1°C.	No detectable live bacteria after 12 minutes of plasma treatment, confirmed by CFU, SEM, and CLSM; plasma outperformed $Ca(OH)_2$, 2% CHX, and $Ca(OH)_2$/CHX (7 days). No significant changes in dentin microhardness or roughness.	Atmospheric pressure cold plasma effectively disinfects 3-week E. faecalis biofilms in root canals within 12 minutes, with no mechanical damage to dentin, offering a promising alternative for endodontic treatment.

Takahashi et al. (2015)	*In vitro*	Ministry of Education, Science, Sports and Culture, Grant-in-Aid for Scientific Research (C) 2008-2010, 20510098; Nihon University Strategic Projects for Academic Research	Coliform biofilms cultured on LB medium in plastic plates (85 mm diameter, 20 mm depth), grown for 24 hours after coating on congealed medium.	Micro low-temperature atmospheric-pressure plasma (LA plasma) using a borosilicate capillary nozzle (50 μm diameter), helium gas (100 ml/min flow), 10 kV pulse voltage at 10 kHz, irradiated for 0, 1, 3, 5, and 10 minutes at an unspecified distance	Plasma irradiation for 5 minutes inactivated a coliform colony area of ~670 mm², with the inactivation region (10–13 mm diameter) larger than the plasma spot (50 μm). Inactivation area increased with irradiation time, attributed to diffusion of O and OH radicals.	Micro LA plasma effectively inactivates coliform biofilms, with O and OH radicals diffusing from the 50 μm nozzle to inactivate a larger region, suggesting potential for oral surgery applications like biofilm sterilization.
Delben et al. (2016)	*In vitro*	Grant 2012/ 20699-6—Sao Paulo Research Foundation grant 2013/21388-7—Sao Paulo Research Foundation (JAD), and grant 2012 GSK/IADR Innovation in Oral Care Award	Single- and dual-species biofilms of *C. albicans* and *Staphylococcus aureus* cultured on acrylic resin discs for 48 hours.	LTP using Kinpen device with argon gas (5 slm flow), 8 W power, 10 mm tip-to-sample distance, applied for 60 seconds with horizontal scanning.	Plasma treatment significantly reduced C. albicans and S. aureus viable counts in single and dual species biofilms compared to negativeand positive controls. CLSM showed increased dead cells throughout biofilm layers, SEM revealed cell wall perforations and flattened biofilm architecture, and fluorescence microscopy confirmed ROS presence in plasma-treated biofilms.	LTP effectively reduces *C. albicans* and *S. aureus* in single- and dual-species biofilms via ROS-mediated cell damage, offering a promising, tissue-safe alternative for oral biofilm treatment.
Preissner et al. (2016)	*In vitro*	No financial support; implants and plasma jet provided by BTI Biotechnology Institute and Neoplas Tools	*Streptococcus mitis* biofilms cultured on microrough titanium dental implants (sandblasted, acid-etched, 2.5 × 13 mm) in BHI broth at 37°C for 84 hours under anaerobic conditions.	kinpen MED device with argon gas (4.3 slm, 4.3 bar), applied at 8 mm tip-to-sample distance for 60 s (TTP60) or 120 s (TTP120), with 60 rpm rotation and 0.22 mm/s (TTP60) or 0.11 mm/s (TTP120) feed under 1% NaCl irrigation.	TTP60 and TTP120 significantly reduced CFU counts (median: 2.19 and 2.2 log CFU/mL) compared to negative control (3.29 log CFU/mL) (p=0.012 and 0.024), with log reduction factors of 2.21 and 1.93, respectively. Diode laser (DL, 980 nm, 2.0 W, 60 s) showed no significant reduction (3.61 log CFU/mL, p=0.674, log reduction 0.59). CLSM and SEM confirmed biofilm presence pre-treatment; fluorescence microscopy showed higher dead cell proportions in TTP groups vs. DL and control. No surface alterations were observed.	TTP effectively reduces S. mitis biofilms on microrough titanium implants, outperforming diode laser, with no surface damage, suggesting potential for peri-implant infection therapy, though clinical studies are needed.

Liu et al. (2017)	*In vitro*	NIH grant no. 5R44DE019041-03	Single-species (*S. mutans*, *S. sanguinis*) and dual-species (*S. mutans* + *S. sanguinis*) biofilms were formed on 48-well plates for 7 days.	Atmospheric non-thermal argon/oxygen plasma brush (3,000 sccm argon, 30 psig; 30 sccm oxygen, 1.4 psig; 10 mA, 0.8 kV), applied 5 mm above biofilms/suspensions for 2 min (biofilms), 15/30 s (A. oris hydrophobicity), or 2 min (A. oris co-aggregation).	Plasma reduced bacterial viability by ≥97% in single- and dual-species biofilms with 75 µL PBS (MTT assay, P<0.05), decreasing to 53–63% with 200 µL PBS and 10–26% with 400 µL PBS. CLSM showed predominantly dead S. mutans (red-stained) post-plasma vs. live (green) in controls. *A. oris* hydrophobicity dropped from 70.7% (control) to 15.3% (30 s plasma, P<0.05). Co-aggregation of A. oris with S. sanguinis was inhibited post-2 min plasma (stable OD vs. decreasing OD in controls, P<0.05)	Non-thermal argon/oxygen plasma effectively reduces oral bacterial biofilm viability and *A. oris* virulence (hydrophobicity, co-aggregation), with efficacy dependent on PBS volume, suggesting potential for dental plaque control.
Matos et al. (2017)	*In vitro*	Sao Paulo State Research Foundation (2013/26145-5 and 2013/08451-1); The Brazilian National Council for Scientific and Technological Development-CNPq (442786/2014-0 and 304908/2015-0), and Fundo de Apoio ao Ensino, à Pesquisa e Extensão da UNICAMP-FAEPEX (2032/15).	Three-species biofilm (*S. sanguinis*, *A. naeslundii*, *F. nucleatum*) cultured on cpTi discs for 16.5 h (early) or 64.5 h (mature).	Non-thermal glow discharge plasma (GDP): 600 s argon/H_2 plasma ablation, 900 s deposition (70% hexamethyldisiloxane, 15% O_2, 15% argon, 13.56 MHz, 100 W), 1800 s pure O_2 plasma (7.5×10^{-2} Torr). Micro-arc oxidation (MAO): 10 min in 0.3 M calcium acetate, 0.02 M glycerophosphate disodium, 300 V, 20°C. Controls: machined (M) and sandblasted (Sb) cpTi.	No significant differences in CFU/mL across groups for early (16.5 h) or mature (64.5 h) biofilms (P>0.05). MAO had higher protein content in early biofilms (P<0.05), but similar carbohydrate levels to controls. SEM and CLSM showed similar biofilm structure, with denser biofilms at 64.5 h.	MAO and GDP treatments increase surface wettability and roughness without promoting increased biofilm formation compared to controls, suggesting potential for dental implants with enhanced osseointegration (via improved pre-osteoblast response) and no elevated peri-implantitis risk. In vitro conditions limit direct clinical applicability.
Ballout et al. (2018)	*In vitro*	Not Specified	*E. faecalis* biofilms cultured for 6 days on standardized curved (28–33°) human root canals (16 mm length, ISO 70 enlargement) from extracted upper central incisors.	Plasma jet (CAP I, kINPen® MED): 60 s indirect irradiation, 4.3 sL/min argon, ~3 mm distance, 5 mL NaCl. Dielectric barrier discharge (CAP II, PlasmaDerm FLEX9060): 60 s direct plasma, 2 mm gap, 10 µs pulses at 10 kV, 300 Hz, 450 mW, 5 mL NaCl. Photodynamic therapy (PDT): 0.05 mL HELBO® Blue Photosensitizer for 30 s, activated by Helbo TheraLite Laser for 60 s, rinsed with 5 mL NaCl. Positive control: 2.5 mL 3% NaOCl + 30 s PUI + 2.5 mL NaOCl. Negative control: 5 mL sterile NaCl for 30 s.	CAP I and CAP II reduced CFUs to median log values of 5.8 in both coronal and apical parts, with CAP I showing slightly better results than CAP II in the coronal part. SEM images revealed dense biofilms in the negative control, while CAP I was more effective than CAP II in disrupting biofilms, though neither achieved complete removal.	CAP I and CAP II reduced CFUs to median log values of 5.8 in both coronal and apical parts, with CAP I showing slightly better biofilm disruption than CAP II, though neither achieved complete removal.

Hafner et al. 2018	*In vitro*	Dr Hafner has given scientific lectures about aPDT and MRONJ sponsored by Bredent Medical GmbH.	*Acinetobacter baumannii* and *Staphylococcus aureus* biofilms foprmed on the surface of surface of tissue culture polystyrene plates for 48 hours.	Plasma ONE device (Plasma MEDICAL SYSTEMS® GmbH, Nievern, Germany) is powered by a 7.2 V/2.6 Ah rechargeable lithium-ion battery with a maximum output of 5 W. It operates at a repetition frequency of 420–1220 Hz, a pulse width of 2.5–10 ms, and a maximum patient current of <100 mA. For all experiments, the device was set to its highest power level (level 5) and used for 120 seconds.	The bactericidal effect of LTP was significantly reduced when the water film over the bacterial culture exceeded 0.3 mm in thickness.	Plasma ONE device, as tested, was not well-suited for moist infection sites, such as the oral cavity, where the depth of water or tissue could impede its effectiveness. Further development of CAPP devices with higher output and different configurations, potentially emphasizing a gas-phase chemistry dominated by RNS, may improve their antibacterial performance in clinical settings.
Nam et al. (2018)	*In vitro*	National Research Foundation of Korea (NRF) funded by the Ministry of Education, Science and Technology (20100010322).	*S. mutans* biofilms on extracted pre-molars.	Dielectric Barrier Discharge (DBD) plasma device featured an alumina tube (4 mm inner diameter) with a stainless steel inner electrode and grounded copper outer electrode. Argon gas flowed at 2 slm, generating plasma at 10 kV between the inner electrode and tube. The enamel surfaces were treated with 15% carbamide peroxide combined with or without LTP for 30 minutes. After treatment, the teeth were rinsed and incubated in artificial saliva for 1 hour, and exposed to *S. mutans* to allow biofilm formation over 24 hours.	Plasma-assisted bleaching with 15% carbamide peroxide was more effective at reducing *S. mutans* adhesion than CP alone, while causing no significant enamel surface changes, making it a safer and less destructive alternative to conventional bleaching.	Plasma-assisted bleaching with 15% CP effectively reduced bacterial adhesion without causing detrimental surface changes to the enamel.
Ulu et al. (2018)	*In vitro*	None.	*Staphylococcus aureus* biofilms were grown for 7 days on SLA titanium discs simulating dental implant surfaces.	"Plasma One" (Plasma Medical Systems, Bad Ems, Germany) was operated with a 2.5 μs pulse width, 1.2 kHz frequency, and 5 W power output for 2 minutes at a fixed 1 mm discharge gap.	LTP demonstrated the highest level of biofilm inactivation, outperforming both laser modes. Surface roughness remained unchanged across all treatments. While contact laser treatment caused localized temperature increases up to 80 °C, CAP maintained a uniform and safe thermal profile.	LTP showed superior antibiofilm activity and thermal safety, making it a promising option for biofilm management on implant surfaces.
Carreiro et al. (2019)	*In vitro*	Titanium discs provided by Singular Implants-Dmr Ind. e Com. de Materiais Odontologicos Ltda, Parnamirim, RN, Brazil.	*P. gingivalis* biofilms cultured on sandblasted, acid-etched titanium grade 4 discs for 5 days.	LTP generated by kINpen device (Leibniz Institute for Plasma Science and Technology-INP, Greifswald, Germany) using argon gas (Ar) at atmospheric pressure, 8 W power at 220 V, 50/60 Hz, 5 slm Ar flow, 7 mm tip-to-sample distance, samples moved horizontally during application for 1 minute (LTP1) or 3 minutes (LTP3).	LTP1 and LTP3 significantly reduced CFU/mL compared to NEGATIVE and GAS controls ($P < 0.001$), though less effective than CHX. Plasma-treated samples showed reduced biofilm, suggesting mechanical removal of dead bacteria. Both treatments exhibited low cytotoxicity and high gingival epithelial viability, with minimal histologic damage.	LTP effectively reduced *P. gingivalis* biofilm on titanium surfaces while being safe for gingival epithelium, with potential for cell repair via VEGF expression, suggesting promise for peri-implant infection treatment.
Hong et al. (2019)	*In vitro and in vivo*	US National Institute of Health (NIH) grants 5R01DE021431 and 5R44DE019041.	*S. mutans* biofilms cultured on sterilized stainless-steel wafers (1 × 1 cm) for 8 hours.	Low-temperature atmospheric plasma brush using 3000 sccm argon and 30 sccm oxygen, powered at 3.0 W (6.0 mA, ~0.50 kV) via DC power supply, applied for up to 2 minutes.	In vitro, 2-minute plasma treatment reduced biofilm by 70% (crystal violet staining), slime production, and DNA quantity (6% of control). Metabolic activity increased (MTT: 273%, aconitase: 446%), enhancing susceptibility to oxidative stress (H_2O_2, paraquat) and ciprofloxacin, with greater CFU reductions than controls. In vivo, plasma-treated rat molars showed 62.5% less decay in upper molars and 31.6% in lower molars, with lower caries rates (31% vs. 52% upper, 67% vs. 88% lower).	Plasma treatment increased biofilm metabolic activity, enhancing susceptibility to antibiotics and host defenses, and reduced caries development in vivo, indicating potential for controlling oral biofilms and preventing caries.

Küçük et al. (2019)	*Clinical*	Scientific and Technological Research Council of Turkey (TÜBITAK), project number 315S274	Subgingival plaque biofilms from periodontal pockets (PD ≥ 5 mm) in 25 systemically healthy periodontitis patients (stage II, III, or IV; grades B), containing red complex bacteria (*Porphyromonas gingivalis, Tannerella forsythia, Treponema denticola*).	Non-thermal atmospheric pressure plasma (NAPP) using Plasma One device (corona discharge, max power 5 W, 420–1220 Hz, 5–10 µs pulse width), applied for 2.5 min per tooth in test group pockets after SRP and saline irrigation; control group received sham application.	The test group (NAPP + SRP) showed greater clinical attachment level (CAL) gain and larger reductions in GI and BOP compared to control. No significant differences in PD or GCF biomarkers were observed. The test group also had greater reductions in key periodontal pathogens and less bacterial recolonization.	Single NAPP application with SRP provided additional CAL gain, reduced GI and BOP, and enhanced elimination of red complex bacteria, delaying recolonization. Repeated applications may further improve outcomes, suggesting NAPP's potential for periodontal pocket decontamination.
Lee et al. (2019)	*In vitro*	National Research Foundation of Korea (NRF) funded by the Korean government (MSIT) (NRF-2016K1A4A3914113).	*Single biofilms of S. mutans, Staphylococcus aureus, Klebsiella oxytoca, Klebsiella pneumoniae formed* on titanium discs for 24 hours.	LTP applied for 2 min (P2) or 10 min (P10) on titanium surfaces, generating reactive oxygen species (ROS) and UV; control (NP) received no treatment.	LTP-treated surfaces became more hydrophilic and had higher surface energy without changing topography. Bacterial adhesion and biofilm formation were significantly reduced, especially for gram-negative bacteria, which also showed more membrane damage.	LTP treatment effectively reduces bacterial adhesion and biofilm formation on titanium, with greater efficacy against gram-negative bacteria due to cell wall differences, suggesting potential for preventing peri-implantitis.
Theinkom et al. (2019)	*In vitro*	Bayern Innovativ GmbH, University Medical Center Regensburg (ReForM B), Deutsche Forschungsgemeinschaft (DFG, grant CI 263/1-3)	*E. faecalis* biofilms cultured for 24, 48, or 72 h on polystyrene petri dishes.	LTP using a surface micro-discharge (SMD) thin-film prototype with ambient air as carrier gas, applied for 1, 3, 5, or 10 min at 3.5 kVpp, 4.0 kHz, 0.5–1 W power; spacer ensured 10 mm (planktonic) or 20 mm (biofilm) distance; positive controls: chlorhexidine (CHX, 0.2% or 2%, 5 min) and UVC radiation (0.005–0.26 J/cm²).	LTP significantly reduced planktonic E. faecalis and biofilm CFU, with greater reductions observed after longer exposure times. For 24 h biofilms, LTP reduced CFU by over 3 $\log_{10}$ after 5 min and more after 10 min. LTP was more effective than CHX at reducing CFU in 48 h and 72 h biofilms. Spectroscopic analysis showed no nucleic acid release post-LTP, indicating no cytoplasmic membrane damage.	LTP effectively reduces E. faecalis in planktonic and biofilm forms, with efficacy comparable to CHX and UVC, particularly at 10 min treatment. Lack of nucleic acid release suggests that cytoplasmic membrane damage is not the primary mechanism, indicating potential for contact-free antimicrobial applications in dentistry and dermatology without inducing resistance.
Nagay et al. (2020)	*In vitro*	São Paulo Research Foundation (FAPESP, grants 2017/03746-8, 2017/19603-8, 2018/09923-6), Coordination for the Improvement of Higher Education Personnel (CAPES, finance code 001)	Mixed biofilms of *C. albicans* and *S. oralis* cultured for 14 days on resinous liner (Coe Soft) specimens.	Non-thermal plasma (NTP) applied to Coe Soft liner surfaces in two forms: (1) SF_6 fluorination (100% SF_6 gas, 16.0 Pa, 50 W, 10 min); (2) HMDSO film deposition (etching with 100% O_2, 15.0 Pa, 50 W, 10 min, followed by deposition with 65.2% HMDSO + 34.8% Ar, 23.0 Pa, 80 W, 1 min 50 s). Control: untreated liner.	LTP treatments increased surface roughness and hydrophobicity, which decreased after 14-day biofilm exposure. SF_6 showed higher sorption than HMDSO, but both maintained surface softness post-biofilm, unlike the control. C. albicans CFU was lower for SF_6 and HMDSO, with no significant difference for S. oralis. SEM revealed different surface structures, and EDS confirmed the presence of F (SF_6) and Si (HMDSO). FTIR showed the HMDSO film with specific bonding, and CLSM highlighted biofilm structure.	LTP treatments (SF_6 and HMDSO) enhance resinous liner surface properties by increasing roughness and hydrophobicity, reducing C. albicans colonization without affecting S. oralis, and maintaining stability post-biofilm exposure. HMDSO films offer superior softness retention and lower sorption, suggesting potential for improving denture liner durability and reducing candida-related stomatitis.
Figueira et al. (2021)	*In vitro*	São Paulo Research Foundation (FAPESP, grants 2018/17707-3,	Single- and multi-species biofilms formed by *S. mutans, S. sanguinis,* and *S. gordonii* on hydroxyapatite discs.	Low-temperature plasma (LTP-argon) jet applied at 10 mm distance from biofilm surface for 30 s, 60 s, and 120 s. Controls: chlorhexidine 0.12% (positive, PC), NaCl 0.89% (negative, NC), and argon flow only (F30, F60, F120).The distance from plasma nozzle to biofilms was 10 mm	LTP treatment significantly reduced biofilm viability for both single- and multi-species biofilms compared to controls ($p < 0.0001$). Notably, S. gordonii was completely eliminated across all exposure times.	LTP treatment is an effective method for reducing cariogenic biofilm viability and may be a promising approach for the development of new dental caries control protocols.

		2019/01676-4, 2019/05856-7)				
Hui et al. (2021) *Novel Technique	*In vitro*	Self-funded by authors and institutions; consumables and titanium discs donated by EMS and Southern Implants (Australia)	Complex human biofilm from saliva of a peri-implantitis patient, grown on titanium discs with machined (Group A) or moderately rough (Group B, alumina-blasted) surfaces, incubated for 96 h at 37°C under anaerobic conditions.	LTP spark plasma pen jet (10 kV, 1.4 Hz pulse, 5 mm spark gap, 5 mm distance from disc surface, max 2°C at jet tip) applied to discs submerged in PBS. Compared to erythritol air abrasion (AA, 7 bar, 20 s, 10 mm distance, 14 µm particles) and combined AA+LTP (COM). Controls: non-contaminated/treated (-ve) and contaminated/untreated (+ve).	LTP significantly reduced biofilm in both groups, with higher reductions observed in Group A compared to Group B. AA and COM treatments also showed strong reductions in biofilm, with significant differences found between treatments in both groups. SEM and laser profilometry revealed no significant surface alterations.	AA with erythritol, with or without LTP, is highly effective for biofilm removal from titanium surfaces, with near-complete decontamination on smooth surfaces. LTP alone is less effective, with no additive effect in COM. All treatments preserve surface topography, supporting potential clinical use for peri-implantitis management, though LTP's in vivo benefits require further study.
Hui et al. (2021)	*In vitro*	Self-funded, material support from EMS and Southern Implants	Human complex biofilm from peri-implantitis patient saliva, grown on Ti implants for 96 h in BHI with 5% sheep blood, 10% saliva, at 37°C, anaerobic, 80 rpm.	LTP: Spark plasma pen jet (1.4 Hz, 10 kV RF voltage, 5 mm distance, in PBS at room temp). AA: EMS Airflow (7 bar, 14 µm erythritol, 20 s per surface, 10 mm distance, 60 mL/min water). COM: AA followed by LTP. Controls: Negative (non-contaminated, AA+LTP-treated), positive (contaminated, untreated).	AA and COM achieved near-complete biofilm removal (94.87% and 95.32%, respectively), while LTP alone removed 52.10%. All treatments significantly reduced biofilm compared to the positive control. SEM showed no surface alterations, with AA and COM effectively clearing biofilm, unlike the positive control	Erythritol, alone or combined with LTP, is highly effective for biofilm removal in a peri-implantitis model without altering Ti surface topography. LTP alone showed moderate efficacy, with no clear additive effect in COM. Further optimization of LTP parameters and liquid medium interactions is needed for clinical use. In vitro model and thin biofilms limit clinical applicability.
Leite et al. (2021)	*In vitro*	São Paulo Research Foundation (2019/05856-7), CAPES (Finance Code 001), CNPq (308127/2018-80)	*C. albicans* biofilms grown on 96-well plates in for 24 or 48 hours.	LTP: Helium DBD jet (32 kHz, 13 kV, 22% duty cycle, 2 slm helium, 1 W, 15 mm distance, 40°C max, applied for 5 min isolated or 2.5 min combined). Antifungals: Nystatin (80 µg/mL) or amphotericin B (5 µg/mL) for 5 min (isolated); lower concentrations for combined treatments. Combinations: Antifungal followed by LTP or vice versa. Controls: Untreated biofilm.	LTP alone (5 min) reduced viable cells more effectively than nystatin or amphotericin B in 24 and 48 h biofilms of both strains. Nystatin only reduced viable cells in P29 strain. Combined treatments (2.5 min LTP and reduced antifungal doses) significantly reduced viable cells, but no synergy was observed. LTP alone outperformed some nystatin combinations for *C. albicans*.	LTP alone is more effective than polyene antifungals against *C. albicans* biofilms, offering a promising alternative for oral candidiasis treatment, especially in antifungal-resistant cases. Lack of synergy in combinations may be due to oxidative stress-induced protective responses in biofilms. Further studies are needed to optimize CAP parameters and investigate resistance mechanisms. In vitro model limits clinical applicability.
Nima et al. (2021)	*In vitro*	São Paulo Research Foundation (2017/03605-2, 2018/06730-2), CAPES (Finance Code 001), CNPq (142138/2017-7), NIH-NIDCR (R03-DE028006-02)	*S. mutans* biofilms grown on resin composite discsfor 24 h.	LTP: Argon plasma jet, handheld unit, 10 mm distance, applied for 30, 90, 120, or 150 s. Controls: Untreated (CON), 2% chlorhexidine (CHX) for 1 min, argon gas only (ARG) for 150 s.	LTP treatments (30–150 s) significantly reduced viable cells (CFU) compared to CON and ARG, with reductions increasing over time. LTP (90–150 s) also significantly lowered metabolic activity compared to shorter exposures. CHX achieved complete CFU and metabolic activity reduction. SEM showed LTP-induced cell damage, and PCR indicated DNA damage in NT.	LTP effectively reduces S. mutans biofilm viability and metabolism in a time-dependent manner, suggesting potential as an intraoral surface-decontamination strategy. CHX remains more effective. In vitro model limits clinical applicability: further studies needed to optimize LTP parameters and assess multispecies biofilms.

Zarif et al. (2021)	*In vitro*	Romanian Ministry of Education and Research (CCCDI-UEFISCDI, 271/2020; PN-III-P2-2.1-PED-2019-4569), INFLPR NUCLEU Program (LAPLAS VI 16N/2019)	Monospecific biofilms of *S. aureus*, *E. faecalis, Escherichia coli*, and *Pseudomonas aeruginosa* grown on HAP pellets (6 mm diameter, 2.2 mm height) in nutritive broth for 24 h at 37°C.	LTP: Planar Dielectric Barrier Discharge (DBDp) with argon gas (99.999% purity, 2000 sccm flow, 20 W, 13.56 MHz RF, 0.8 mm distance) applied for 3 min. Groups: Untreated HAP (HAP_i), fluoride gel only (HAP_g, 1450 ppm potassium fluoride, 3 min), plasma only (HAP_DBDp), plasma + gel (HAP_DBDp_g). Gel applied with cotton swabs, removed after 3 min without rinsing.	Bacterial viability at 12 h was significantly reduced in the plasma + gel-treated Hydroxyapatite compared to untreated Hydroxyapatite (HAP_i), with up to a 6.7 log CFU/mL reduction for S. aureus. Plasma-only-treated Hydroxyapatite (HAP_DBDp) showed moderate reductions, while fluoride gel-only-treated Hydroxyapatite showed the lowest. Biofilm formation was significantly reduced in both plasma + gel-treated Hydroxyapatite and plasma-only-treated Hydroxyapatite.	DBDp enhances fluoride retention in HAP, likely via surface hydrophilization, improving antibacterial and antibiofilm effects against Gram-positive and Gram-negative bacteria. HAP_DBDp_g shows synergistic effects, surpassing fluoride gel alone.
Asnaashari et al. (2022)	*In vitro*	Laser Application in Medical Sciences Research Center, Shahid Beheshti Medical University	*E. faecalis* biofilms grown in single-rooted human teeth for 21 days.	LTP: Helium/oxygen plasma jet (95% He, 5% O_2, 8 kV, 14 kHz, 10 mm distance) applied for 10 min. Groups: Negative control (no biofilm), positive control (4% methylcellulose, 12 days), calcium hydroxide (2 g/2 mL, 12 days), TAP (10 mg/mL ciprofloxacin, metronidazole, minocycline with 4% methylcellulose, 12 days), plasma (10 min), plasma positive control (untreated biofilm).	LTP reduced CFU by 11.6% compared to its control. SEM confirmed biofilm disruption in plasma-treated canals (open dentinal tubules, irregular biofilm structure) vs. thick, mature biofilm in positive control. Negative control showed no biofilm.	LTP significantly reduced biofilm but was less effective than triple antibiotic paste. LTP's potential as a safe disinfectant for regenerative endodontics is noted, given no stem cell toxicity, but it requires optimization for complete biofilm elimination. In vitro model limits clinical applicability; further studies needed for other bacteria and plasma parameters.
Hong et al. (2022)	*In vitro*	Nanova Inc., Curators of University of Missouri	*P. gingivalis* biofilms grown on 316L stainless steel coupons for 3 or 5 days	LTP brush with pure argon (3000 sccm) or argon + 1% oxygen (3000 sccm Ar + 30 sccm O_2), 6 mA, 37°C, scanned for 1, 2, or 5 min. Biofilms rinsed with PBS, treated, and assessed via MTT assay, CFU counting, and CLSM. Recovery tested after 2 days in TSB with amoxicillin (1/32, 1/16 µg/mL), H_2O_2 (0.002–0.008 wt%), or paraquat (12.5–50 mM).	LTP (argon + 1% O_2, 5 min) significantly reduced 3-day biofilm by 83.38% (MTT) and 2.7 log CFU, and 5-day biofilms by 81.93% (MTT) and 4.07 log CFU. CLSM confirmed widespread bacterial death after 5 min. LTP combined with amoxicillin, H_2O_2, or paraquat showed additional reductions in CFU compared to untreated controls.	LTP effectively reduced P. gingivalis biofilms, with argon + 1% O_2 outperforming pure argon. Efficacy was higher for 3-day vs. 5-day biofilms. LTP increased biofilm susceptibility to amoxicillin, H_2O_2, and paraquat, enhancing host defense. While complete biofilm elimination was not achieved, LTP shows promise as adjunct therapy for periodontitis.
Kamionka et al. (2022)	*In vitro*	German Ministry of Education and Research (BMBF, grant no. 13N9779), Ministry of Education, Science and Culture of Mecklenburg-Western Pomerania, European Social Fund (Grant number: AU 11 038; ESF/IV-BM-B35-0010/13)	*S. mutans and P. gingivalis* biofilms on both sandblasted and anodized titanium surfaces.	kINPen08 plasma jet (Leibniz Institute, Germany) using argon gas (99.999%) at 5 slm flow, 1 MHz frequency, 2–6 kVpp, 3.5 W max power, 10 mm plasma plume, 46–63°C temperature; applied at 5 mm distance for 9 min per disc side (60 s at 9 spots) using a computer-controlled x/y/z table. Air-polishing with glycine (APG, 25 µm) or erythritol + 0.3% CHX (APE, 14 µm) powders for 90 s per side (15 s at 4 spots, 30 s meandering) at 4 ± 1 mm distance, 65° angle.	CAP alone reduced biofilm fluorescence by 15.4% (sandblasted) and 25.2% (anodised) on day 0. APG and APE reduced fluorescence by 77.9–92.5% on day 0, with APE + CAP showing slightly better results than APG + CAP. On day 5, APG and APE showed regrowth (55.1–86.0% reduction on sandblasted, 31.0–35.1% on anodised), while APG + CAP and APE + CAP maintained 77.2–90.6% reduction, comparable to sterile controls. Sandblasted surfaces showed better cleaning than anodised.	Combined air-polishing and CAP treatment significantly enhances biofilm removal and inactivation, achieving near-sterile surfaces, especially on sandblasted/acid-etched titanium, offering a promising approach for peri-implantitis treatment.

Matthes et al. (2022)	*In vitro*	German Ministry of Education and Research (Grant numbers 13N14478, 13N14479 and 13N14480).	Subgingival plaque from a periodontally diseased volunteer, cultivated on sandblasted/acid-etched titanium discs in 96-well plates for 7 days.	kINPlas plasma jet (Leibniz Institute, Germany) using argon gas (99.999%) at 2.3 slm flow, 0.95 MHz frequency, 2–3 kVpp, 1.6 W max power; applied at 5 mm distance for 70 s per disc side (meandering motion, 2 mm/s) using a computer-controlled x/y/z stage. Water jet (WJ) with sterile saline (0.9%) at 72 ± 2 ml/min, 0.5 mm distance, 30 s per side (meandering). Curette and cotton swab (CC) with gentle strokes and PSS-soaked swab for 30 s per side, rinsed with 1 ml PSS.	Combining a water jet with cold atmospheric plasma (WJ + CAP) most effectively removed biofilm from rough titanium discs, preserved surface integrity, enhanced cell adhesion, and supported osteoblast-like cell growth, showing superior decontamination and biocompatibility compared to curette and cotton swab methods, with or without CAP.	WJ + CAP achieves superior biofilm removal and biologically acceptable surfaces compared to CC + CAP, WJ, or CC alone, with high osteoblast coverage and minimal microbial regrowth, offering a promising non-destructive approach for peri-implantitis treatment.
Panariello et al. (2022)	*In vitro*	National Institute of Dental and Craniofacial Research (NIH/NIDCR—1R21DE028929-01).	Newly formed (24 h), intermediate (3 days), and mature (7 days) peri-implant-related biofilms formed on titanium surfaces. The multispecies biofilm consisted of *A. naeslundii, P. gingivalis, S. oralis, and V.dispar.*	kINPen MED operated with Argon gas. System power: 8 W at 220 V, 50/60 Hz. Working distance: 5- and 10-mm nozzle-to-sample. Application time: 1, 3 and 5 minutes.	LTP treatment significantly reduced the CFUs of all bacteria species compared to the negative control which received no treatment in all biofilm periods and treatment conditions ($p \leq 0.016$), and CLSM corroborated these results.	LTP application effectively reduces peri-implantitis-related multispecies biofilms on titanium surfaces in vitro.
Matthes et al. (2023)	*In vitro*	German Federal Ministry of Education and Research (BMBF; grant numbers 13N14478, 13N14479, and 13N14480, to T.K.) and BMBF infrastructure funding (grant number 03Z22Di1, to S.B.).	Seven-day-old multispecies biofilms generated from subgingival plaque of a single periodontally diseased individual, cultivated on titanium implants and discs in Schaedler Broth, with daily medium replacement.	Cold atmospheric pressure plasma (CAP) jet (periINPlas, Leibniz Institute) operated with argon gas at 0.95 MHz, 2–3 kVpp, 1.6 W max input DC power, 2.3 slm flow rate; implants treated for 120 s with hand-held CAP at 20–90° angles; titanium discs treated for 58 s/side using a computer-controlled stage at 5 mm distance with meandering motion.	Waterjet + CAP showed the highest decontamination efficacy (mean score 0.5, 95% reduction vs. cotton gauze), reducing microorganisms and organic deposits to <0.3% on implants, comparable to positive controls; Waterjet alone outperformed cotton gauze (mean score 4.0 vs. 9.2); CAP reduced chemokine/cytokine levels (e.g., IL1β, IL6, TNFα) and did not promote leukocyte activation.	Combined Waterjet and CAP treatment effectively removed biofilms from titanium implants, outperformed cotton gauze, and reduced inflammatory potential without inducing non-specific immune activation, suggesting potential for peri-implantitis treatment.
Avukat et al. (2023)	*In vitro*	Advanced Material Technologies Application and Research Center.	*C. albicans* biofilms formed on polished PMMA discs.	Helium plasma treatment via RF sputter (Dressler Cesar 136 RF Generator) with helium/oxygen gas mixtures (100:0, 90:10, 85:15, 80:20) at 30 W RF power, 2 sccm gas flow, 120 s duration, 10 cm cathode-sample distance; groups: G I (untreated), G II (80% He), G III (85% He), G IV (90% He), G V (100% He).	Helium plasma-treated groups (G II–G V) showed significant reductions in C. albicans viability and biofilm formation compared to control (G I); MTT assay: G V< G IV < G III < G II < G I; CV assay: G V < G IV (2.29 ± 0.10) < G III < G II < G I; SEM confirmed reduced adhesion in treated groups, with G V showing the least biofilm; surface roughness (0.08–0.2 µm) unchanged.	Helium plasma treatment, particularly at higher concentrations, effectively reduced C. albicans biofilm formation and viability on PMMA surfaces, suggesting a promising strategy to prevent denture stomatitis by modifying surface properties without altering mechanical characteristics.

Canullo et al. (2023)	*Clinical (randomized proof-of-concept)*	No Funding to Declare	Diverse peri-implant bacterial biofilms (283 species, including early/late colonizers and pathogens) and less diverse fungal biofilms (11 species, mainly Malassezia restricta) on titanium healing abutments, assessed 2 months post-implantation.	Argon plasma pre-treatment (PT) in a plasma reactor (Diener Electronic GmbH) at 75 W power, -10 MPa pressure, 12 min duration, applied to machined (smooth) and rough (ultrathin threaded microsurface) abutments before connection; groups: PT (n=18, 9 machined/9 rough), NPT (n=18, 9 machined/9 rough).	PT significantly reduced plaque accumulation and inflammation (lower PI and BOP) compared to NPT, with higher abundance of early colonizers (e.g., Streptococcus mitis, S. gordonii) and lower presence of late colonizers (e.g., Neisseria oralis); no significant effect on peri-implant soft tissue phenotype (PISP) or microbial diversity; PT showed co-occurrence of *S. mitis* with *Rothia dentocariosa* and co-exclusion with pathogens like *Parvimonas micra*.	Argon plasma pre-treatment of healing abutments reduced biofilm accumulation and inflammation, promoting a less advanced biofilm state, but did not significantly alter PISP, suggesting a promising approach for improving peri-implant health.
Ji et al. (2023)	*In vitro*	National Research Foundation of Korea (NRF) grant (No. NRF-2020R1C1C1005683, NRF-2020R1I1A1A01073015)	*S. mutans* and *P. gingivalis* biofilms cultured on anodized TiO_2 nanotubes i*n artificial saliva, assessed after 24 h (S. mutans*) and 48 h (*P. gingivalis*).	Atmospheric pressure plasma (Ar + 1% O_2, 300 W, 120 s, 10 L/min flow rate) using PGS-200 generator (Expantech); applied to TiO_2 nanotubes with no heat treatment (H0), 400°C heat-treated (H400, anatase), or 600°C heat-treated (H600, anatase/rutile mix).	Plasma treatment significantly reduced *S. mutans* biofilm thickness across all groups, with no significant effect on *P. gingivalis*; plasma increased hydrophilicity (lower contact angles) and altered surface chemistry (reduced C-H/C-C, increased O-H/TiO_2 bonds) without changing nanotube structure.	Plasma treatment further reduced *S. mutans* adhesion, likely due to increased hydrophilicity, suggesting potential for improving dental implant surfaces to mitigate peri-implantitis.
Zhu et al. (2023)	*In vitro*	National Natural Science Foundation of China (No. 81901064) and Beijing Hospitals Authority Youth Programme (No. QML20210304).	*E. faecalis* biofilm cultured for 7 days on human dentin blocks and confocal dishes.	Cold atmospheric pressure plasma-loaded microbubbles (PMBs) fabricated via modified emulsification with plasma gas (Span 60, NaCl, Tween 80, PBS, PEG-4000); PMBs (1-2 μm diameter) at 10^7 mL^{-1} and 10^8 mL^{-1}, combined with ultrasound (3 W/cm², 1 MHz, 50% duty cycle); groups: PBS, 2.5% NaOCl, 2% CHX, PMBs (10^7 mL^{-1}) + US, PMBs (10^8 mL^{-1}) + US; treatment for 5 min.	PMBs with ultrasound significantly reduced viable bacteria and removed biofilm on confocal dishes and dentin surfaces, especially in dentin tubules, outperforming 2.5% NaOCl and 2% CHX in tubule disinfection; higher PMB concentration (10^8 mL^{-1}) was more effective; NaOCl removed surface biofilm but left tubule residues; CHX killed bacteria but left biofilm matrix; PMBs maintained dentin microhardness and roughness.	PMBs with ultrasound effectively removed *E. faecalis* biofilm, particularly in dentin tubules, with superior performance compared to NaOCl and CHX, and maintained mechanical safety, suggesting potential as an innovative endodontic disinfection method.
Figueira at al. (2024)	*In vitro*	Sao Paulo Research Foundation/FAPESP (Process# 2018/17707-3, 2019/01676-4 and 2019/05856-7).	Multispecies cariogenic biofilms consisting of *C. albicans, L. casei,* and *S. mutans*, along with single-species biofilms of *C. albicans* and *L. casei* cultured on hydroxyapatite.	kINPen09™ (Leibniz Institute for Plasma Science and Technology, INP, Germany), operated with argon gas at 8 W (220 V, 50/60 Hz). Biofilms formed on hydroxyapatite discs were exposed to the plasma jet at a fixed distance of 10 mm from the device tip to the biofilm surface, with continuous treatment durations of 30, 60, and 120 seconds.	LTP significantly reduced multispecies biofilm viability from 30 seconds of application. In single-species biofilms, *L. casei* showed greater reduction than with chlorhexidine or saline, while *C. albicans* responded significantly at 60 and 120 seconds, indicating a time-dependent effect for both species.	LTP is a potential mechanism in treating dental caries by being an effective anti-biofilm therapy of both single and multispecies cariogenic biofilms.
Haude et al. (2024)	*In vitro*	German Ministry of Education and Research (BMBF, grants 13N9779, 13N14478), the Ministry of Education, Science and Culture of Mecklenburg-Western Pomerania, the	Subgingival plaque from deep pockets of a periodontally diseased volunteer cultured for 24 hours on titanium discs	Two plasma devices (kINPen® 09- CAP09 and kINPen® MED- CAPmed) were operated at a frequency of approx. 1 MHz, with argon as the carrier gas, and a plasma plume length of 10 mm. The kINPen® MED used a duty cycle of 50% and a repetition frequency of 2.5 kHz. Plasma was applied for 30 seconds, 60 seconds, and 120 seconds. The distance between the plasma nozzle and the surface of the biofilm was set to 10 mm.	While CAP09 and CAPmed alone did not effectively remove biofilm, glycine powder (AP) treatment detached the biofilm completely, though it regrew to baseline levels after 5 days. The combination therapies (AP + CAP09 and AP + CAPmed) achieved complete biofilm removal immediately after cleaning, but biofilm regrowth occurred on 50% of the discs after 5 days.	The combination of AP and plasma treatment, however, was more effective in addressing these inaccessible areas, leading to a more sustained biofilm removal.

		European Union (European Social Fund, grants AU 11 038; ESF/IV-BM-B35-0010/13), and Open Access funding organized by Projekt DEAL.				
Panariello et al. (2024)	*In vitro*	National Institute of Dental and Craniofacial Research (NIH/NIDCR—1R21DE028929-01).	Mature 14- and 21-day-old peri-implant-related biofilms formed on titanium surfaces. The multispecies biofilm consisted of *A. naeslundii, P. gingivalis, S. oralis,* and *V.dispar.* The impact of LTP was investigated on reconstituted oral epithelia (ROE) contaminated with *P. gingivalis.*	kINPen MED operated with Argon gas. System power: 8 W at 220 V, 50/60 Hz. Working distance: 5 and 10 mm nozzle-to-sample. Application time: 1, 3 and 5 minutes.	A 1-minute LTP exposure at both distances significantly reduced bacterial counts ($p < 0.017$), with minimal cytotoxicity ($p = 0.038$) and no negative impact on cell viability ($p \geq 0.05$). Histology confirmed tissue safety.	LTP effectively disinfects mature biofilms on titanium while maintaining biocompatibility with oral epithelial tissue, supporting its potential as an adjunctive peri-implantitis treatment.
Puca et al. (2024)	*In vitro*	NextGeneration EU—MUR, Fondo Promozione e Sviluppo, DM 737/2021 and National Research Foundation of Korea (2021R1A6A1A03038785).	*S. mutans* and a mixture of microorganisms isolated from the saliva of a patient with periodontitis formed on the surface of tissue culture polystyrene plates for 48 hours.	The LTP device used atmospheric air and consisted of high-voltage and ground electrodes, a power supply, and dielectric materials. Air flow was set at 2 L per minute, with a 2 mm discharge gap. The plasma jet, applied 6 mm from the biofilm for 60 seconds, was generated by a stainless-steel needle (1.20 mm by 0.27 mm) with a perforated outer electrode.	LTP treatment significantly reduced CFUs and XTT levels after just 60 seconds. CLSM analysis showed LTP effectively killed microorganisms within the biofilm and reduced its matrix thickness. Cytotoxicity tests confirmed that LTP had minimal harm to human gingival fibroblasts.	LTP effectively reduced preformed biofilms developed by *S. mutans* and a mixed microbial community derived from saliva.
Lim et al. (2025)	*In vitro*	Trans-disciplinary Research Grant Scheme (TRGS/1/2020/UM/02/2/3) from Malaysia's Ministry of Education.	*S. mutans* biofilms formed on polystyrene microtiter plate wells and incubated for 48 hours.	Hollow cylindrical glass tube (1.5 mm inner diameter, 3 mm outer diameter) with a high-voltage electrode and a copper sheet as the grounded electrode. The plasma plume extended 4 mm from the tip, powered by an APVM500 plasma generator. LTP was applied 7 mm from the biofilm surface for 0 (control), 1, 6, 10, and 15-minute durations.	The highest reduction in biofilm was achieved after 15 minutes of LTP exposure, resulting in a 3.08 log reduction of *S. mutans* cells. A 10-minute treatment showed a 1.79 log reduction, while 1 minute of treatment led to only a 0.23 log reduction.	The effectiveness of LTP treatment was strongly correlated with the duration of exposure. These results demonstrate LTP's potential as a promising, non-thermal alternative to traditional antimicrobial methods.
Morais et al. (2025)	*In vitro*	Coordination for the Improvement of Higher Education Personnel - CAPES (ROR identifier: 00x0ma614).	*C. albicans* biofilms formed on the surfaces of titanium discs for 48 hours.	Dielectric barrier discharge (DBD) system, powered by a 13 kV discharge at a frequency of 600 Hz, and a helium gas flow rate of 1.5 L/min. The plasma jet was applied at a distance of 10 mm from the surface.	Notable reduction in *C. albicans* CFUs and decrease in pseudohyphae formation after LTP application.	This study highlights the potential of LTP as an effective non-thermal method for reducing fungal contamination on titanium alloy surfaces used in medical and dental implants.

Table S3: List of excluded studies and reasons for exclusion.

Study	Reason
1. Effect of tooth bleaching using cold plasma (Magnetic Fields) combined with a tooth bleaching gel containing strawberry juice<br>Nam et al., 2024	Unrelated to oral biofilms outcomes
2. Evaluation of the Promotion for Antibacterial Activity on Cibotium barometz J. Smith with Low-Temperature Plasma from Electric Currents and Magnetic Fields<br>Nam et al., 2024	Not in English
3. Inactivation kinetics study of an atmospheric-pressure cold-plasma jet against pathogenic microorganisms<br>Sedghizadeh et al., 2012	Unrelated to oral biofilms outcomes
4. Biofilm models for the evaluation of dental treatment<br>Eick, 2021	Book chapter
5. Effects of Nonthermal Atmospheric Pressure Plasma Jet on Human Dental Pulp Stem Cells<br>Mousavi et al., 2021	Unrelated to oral biofilms outcomes
6. Atmospheric cold plasma interactions with microbiological risks in fresh food processing<br>Patange et al., 2019	Unrelated to oral biofilms outcomes
7. Atmospheric cold plasma as a tool for microbiological control<br>Ziuzina, 2015	Unrelated to oral biofilms outcomes
8. Atmospheric cold plasmas for biofilm inactivation: does biofilm extracellular matrix limit the bactericidal process?<br>Marchal et al., 2013	Unrelated to oral biofilms
9. Ozone: a new revolution in dentistry<br>Nagarakanti et al., 2013	Unrelated to Low-temperature plasmas outcomes
10. Low temperature growth of thin film coatings for the surface modification of dental prostheses<br>Mandracci et al., 2008	Unrelated to oral biofilms outcomes
11. The photodynamic therapy on *Streptococcus mutans* biofilms using erythrosine and dental halogen curing unit<br>Lee et al.,2012	Unrelated to Low-temperature plasmas outcomes
12. Deactivation of Oral Bacteria Using an Atmospheric Plasma Brush<br>Yang et al.	Conference abstract
13. Oral microbiology and immunology<br>Lamont et al., 2020	Book chapter
14. Biodecontamination of Plastic and Dental Surfaces with Atmospheric Pressure Air DC Discharges	Conference abstract
15. Advanced Plasma Technology<br>16.1 Introduction<br>Stoffels, 2008	Book chapter

16. Influence of daily immersion in denture cleanser on multispecies biofilm<br>Lucena-Ferreira et al., 2014	Unrelated to Low-temperature plasmas outcomes
17. In vitro activity of superoxide water on viability of *Enterococcus faecalis* biofilm on root canal wall<br>Savadkouhi et al., 2021	Unrelated to Low-temperature plasmas outcomes
18. Cold atmospheric plasma, the removal of blood from steel and its effect on staphylococcal biofilm formation. A pilot study<br>Fallon et al., 2020	Unrelated to oral biofilms outcomes
19. Biofilm and saliva affect the biomechanical behavior of dental implants<br>Bordin et al.,2015	Unrelated to Low-temperature plasmas outcomes
20. Biofilm and cell adhesion strength on dental implant surfaces via the laser spallation technique<br>Boyd et al., 2021	Unrelated to Low-temperature plasmas outcomes
21. The effect of Cold Atmospheric Plasma (CAP) on the molecular mechanism of wound healing<br>Shome, 2020	Unrelated to oral biofilms outcomes
22. Effects of chlorhexidine on a tongue-flora microcosm and VSC production using an in vitro biofilm perfusion model<br>Greenman et al., 2008	Unrelated to Low-temperature plasmas outcomes
23. Micro-Biocidal Activity of Yeast Cells by Needle Plasma Irradiation at Atmospheric Pressure<br>Kurumi et al., 2014	Conference abstract
24. Influence of anguled implant position on bone strains and stress<br>Abboud, 2013	Unrelated to low-temperature plasmas outcomes
25. Evaluation of Antibiofilm Efficacy of Different Nanoherbal Medications on Multispecies Bacterial Infected Root Canals: An Invitro study<br>Aboammo et al., 2018	Unrelated to Low-temperature plasmas outcomes
26. Twice-daily red and blue light treatment for *Candida albicans* biofilm matrix development control<br>Silveira et al., 2019	Unrelated to Low-temperature plasmas outcomes
27. Plasma-initiated graft polymerization of carbon nanoparticles as nano-based drug delivery systems<br>Liu et al., 2022	Unrelated to oral biofilms outcomes
28. On-Chip monitoring of fungal biofilms using a Lab-on-a-chip<br>Richter, 2008	Book chapter
29. Measurement of electro-osmosis as a method for electrokinetic surface analysis<br>Quartz, 1999	Book chapter
30. Osteoblast growth, after cleaning of biofilm-covered titanium discs with air-polishing and cold plasma.<br>Matthes et al., 2017	Unrelated to oral biofilms outcomes
31. Cold atmospheric plasma in combination with mechanical treatment improves osteoblast growth on biofilm covered titanium discs.	Unrelated to oral biofilms outcomes

Duske et al., 2015	
32. Colonization by *Staphylococcus aureus* of Nano-Structured Fluorinated Surfaces, Formed by Different Methods of Ion-Plasma Technology.<br>Elinson et al., 2016	Unrelated to oral biofilms outcomes
33. Antibacterial potential of silver and zinc loaded plasma-electrolytic oxidation coatings for dental titanium implants.<br>Paiwand et al., 2025	Unrelated to Low-temperature plasmas outcomes
34. Immobilization of quaternary ammonium based antibacterial monomer onto dentin substrate by non-thermal atmospheric plasma.<br>Liu et al., 2019	Unrelated to Low-temperature plasmas outcomes
35. Effect of argon plasma abutment activation on soft tissue healing: RCT with histological assessment.<br>Canullo et al., 2024	Unrelated to oral biofilms outcomes
36. Coated vs uncoated implants: bone defect configurations after progressive peri-implantitis in dogs.<br>Madi et al., 2014	Unrelated to low-temperature plasmas
37. Effect of modified nonequilibrium plasma with chlorhexidine digluconate against endodontic biofilms in vitro.<br>Du et al., 2013	Unrelated to Low-temperature plasmas outcomes
38. Adsorption behavior of antimicrobial peptide histatin 5 on PMMA.<br>Yoshinari et al., 206	Unrelated to Low-temperature plasmas outcomes
39. Plasma in Dentistry: Brief History and Current Status.<br>Gherardi et al., 2018	Review article
40. Advancing antimicrobial strategies for managing oral biofilm infections.<br>Jiao et al., 2019	Review article
41. Comprehensive biomedical applications of low temperature plasmas.<br>Duarte & Panariello, 2020	Review article
42. Effects of Cold Atmospheric Pressure Plasma on *Streptococcus mutans* isolated from Dental Caries<br>Ali et al., 2024	Conference abstract
